# Modeling and control of Chikungunya with chronic infection

**DOI:** 10.1016/j.idm.2025.12.002

**Published:** 2025-12-10

**Authors:** Yan Wang, Huan Ma, Qian Yan, Zhichun Yang

**Affiliations:** a School of Mathematical Sciences, Chongqing Normal University, Chongqing, 401331, PR China; b School of Mathematics and Statistics, HuBei Minzu University, Enshi, Hubei, 445000, PR China

**Keywords:** Chikungunya, Chronic infection, Stability analysis, Optimal control, Cost-effectiveness, 2020 MSC 34D23, 49J15, 92D30

## Abstract

Recognized globally as a major public health concern in the tropics and subtropics, Chikungunya fever also poses a potential epidemic risk in areas of China such as Guangdong Province, where suitable mosquito vector habitats exist. Based on a Chikungunya fever outbreak in Shunde District, Foshan City, this study develops a dynamical model incorporating a chronic infection stage. We derive *R*_0_ and perform a thorough stability analysis of all equilibria. Using daily reported case data from Shunde District, model fitting yields estimates for three key transmission parameters (*β*, *ρ*_1_, *ρ*_2_), the total mosquito population (*T*_*v*_), and the initial number of infected mosquitoes (*I*_*v*_(0)). Sensitivity analysis identifies that the primary positive and negative parameters on disease transmission are mosquito biting rate (*β*) and mosquito mortality rate (*ϵ*_*v*_), respectively. Accordingly, five types of intervention measures are designed: personal protection, screening and detection, treatment of acute patients, management of chronic cases, and mosquito vector control measures. Based on these findings, we formulate a control framework to optimize intervention strategies. Numerical simulations not only validate the global asymptotic stability of the disease-free equilibrium when *R*_0_ < 1 and that of the endemic equilibrium when *R*_0_ > 1, but also assess the effectiveness of different control strategies. Strategy A, which emphasizes personal protection, emerges as the most economically efficient option in the cost-effectiveness analysis. It not only effectively interrupts virus transmission but also optimally reduces the burden of chronic cases, thereby offering a scientifically sound and economically feasible approach for public health resource allocation.

## 1 Introduction

Chikungunya virus (CHIKV), a single-stranded RNA Alphavirus within the Togaviridae family (Tang et al., 2023), is the etiological agent responsible for Chikungunya fever. This arboviral disease is primarily spread by Aedes mosquitoes and has a global distribution. Its cardinal clinical feature is characterized primarily by an acute high fever followed by intense arthralgia (Ortigoza et al., 2020). Chikungunya fever is rarely life-threatening, with a fatality rate of approximately 0.1 % (Pialoux et al., 2007). However, about 30–40 % of patients may develop chronic arthritis that persists for months or even years, resulting in significant loss of workforce capacity (Flandes et al., 2024; Milligan et al., 2019; Puntasecca et al., 2021; Van Aalst et al., 2017). The persisting sequelae and chronic symptoms of the disease place a substantial economic and societal burden on both individuals and communities (Cao et al., 2025). Specifically, in Brazil's private healthcare system, the per capita hospitalization cost for patients with chronic symptoms reaches as high as USD 2,400, accompanied by prolonged work absenteeism (Andrade et al., 2022). In India, Chikungunya outbreaks have resulted in an estimated productivity loss ranging from USD 2.57 million to USD 4.69 million due to absenteeism (Costa et al., 2023). The emergence of Chikungunya poses a serious public health threat, characterized by increased strain on healthcare systems, significant direct economic costs, as well as the deterioration of the socioeconomic order in endemic areas (Simon et al., 2011). Thus, the chronic stage represents a major contributor to the long-term disease burden of Chikungunya, making it imperative to incorporate this phase in relevant models.

Chikungunya fever is primarily distributed in tropical and subtropical regions. However, driven by factors such as climate change, globalization, and urbanization, the risk of importation and re-emergence of the virus into new geographical areas continues to increase (Kang et al., 2024). Since its initial identification in 1952, local transmissions have occurred in over 110 countries across the globe. Southern China, particularly Guangdong Province, is recognized as a high-risk area for both imported and locally transmitted cases of Chikungunya fever, due to the widespread distribution of *Aedes mosquito* vectors. The high population density and subtropical climate in Guangdong provide conducive conditions for mosquito proliferation, further amplifying transmission risk. In 2010, the first local outbreak in China was reported in Dongguan. Another outbreak occurred in Guangzhou in 2019. Most recently, on July 8, 2025, a locally acquired outbreak triggered by an imported case was detected in Shunde District of Foshan City, Guangdong Province. The epidemic subsequently spread to the entire Foshan municipality, demonstrating the disease's continuing public health threat to southern China.

Within epidemiology, mathematical models have become a core tool for analyzing epidemic patterns and predicting transmission trends, owing to their ability to quantitatively analyze complex transmission processes. A early compartmental model for Chikungunya transmission was constructed by Dumont et al. (2008) based on the outbreak in Réunion Island. Moulay et al. (2011, 2012) later proposed a model incorporating time-varying control. Through theoretical and numerical analysis, they demonstrated the cost-effectiveness advantage of dynamically adjusting control efforts in response to epidemic dynamics. In recent years, the research on Chikungunya fever has attracted extensive efforts from scholars exploring its transmission mechanisms and control strategies from diverse perspectives. Stochastic mathematical modeling of Chikungunya transmission (Cao et al., 2025; Fahimi et al., 2025), has been employed to analyze dynamic behaviors such as disease persistence and extinction through theoretical derivation. Furthermore, several studies have characterized the spread of Chikungunya via fractional-order differential equations (Jain & Chalishajar, 2023; Yangla et al., 2023), systematically investigating the advantages of fractional operators in capturing transmission characteristics and their application in optimizing public health strategies. Some studies have designed optimal intervention strategies by integrating antibody regulation or temperature effects with multiple control measures (El Hajji, 2021; Lusekelo et al., 2023). Another studies have developed models incorporating time-varying parameters, impulse control, nonlocal periodic reaction-diffusion, and periodic models accounting for temperature and rainfall influences, respectively (Liu & Stechlinski, 2015; Liu et al., 2020a, 2020b; Li & Zhao, 2024). These works explore how climatic factors, spatial heterogeneity, and asymptomatic infections influence the transmission mechanisms, epidemic characteristics, and control effectiveness of Chikungunya fever. Feng et al. (2019) and Wang et al. (2022) developed dynamic models from the perspective of CHIKV mutation and analyzed the impact of viral mutations, seasonal temperature variations, and other factors on transmission through real-world case studies and numerical simulations. Additionally, some models have incorporated factors such as chronic infection, asymptomatic infection, or climate influences to study control strategies in specific regions (González-Parra et al., 2019; Helikumi et al., 2022; Trentini et al., 2018; Vázquez-Peña et al., 2023). These studies provide a valuable foundation to comprehend the dynamics of CHIKV transmission and to develop effective, science-based control measures.

However, there is currently limited research focusing on the disease status of chronic infected individuals. In fact, viewing infectious disease dynamics as a multi-state process encompassing asymptomatic infection, a highly contagious acute phase, and a prolonged chronic phase has been established as a key theoretical framework for modeling such complex pathologies. In particular, research (Guo et al., 2012; Guo & Li, 2011; Liu & Li, 2021; Sanz-Lorenzo et al., 2025; Song et al., 2017) demonstrated that accurately characterizing the heterogeneity among individuals across different infection states and their transition dynamics is central to understanding the overall dynamics and long-term impact of an epidemic.

Therefore, although chronic Chikungunya patients are non-infectious, the preceding discussion underscores that their impact on society cannot be overlooked. Neglecting this phase would lead to an underestimation of the pandemic's overall socioeconomic impact and introduce bias into the cost-effectiveness evaluation of public health interventions. Incorporating the chronic stage into model not only expands the analytical scope from mere transmission dynamics to disease burden assessment but also provides critical insights for public health decision-making, thereby guiding rational resource allocation and promoting the design of optimized intervention strategies. Consequently, this paper addresses the Chikungunya fever outbreak in Foshan and aims to develop a model that incorporates chronic infection to simulate the bidirectional human-mosquito transmission process. Designed with a dual purpose, the model aims to replicate CHIKV transmission patterns and quantify the associated disease burden more comprehensively. Following the assessment of system's stability properties, parameter estimation is conducted using daily reported case data from Shunde District of Foshan, identifying key sensitive parameters influencing Chikungunya spread. Based on the findings, relevant control measures are proposed, and numerical simulations along with cost-effectiveness evaluation of intervention strategies are performed.

The paper proceeds as follows. The mathematical model is formulated and its stability is examined in Section 2. The subsequent section addresses the estimation of parameters and presents numerical results of stability properties. Turning to sensitivity analysis, Section 4 identifies the key parameters influencing *R*_0_ and disease spread. The optimal control framework is then analyzed in Section 5. Section 6 simulates various control measures and evaluates their economic efficiency. The final section offers a general conclusion and discussion of the study.

## 2 Mathematical model and stability analysis

### 2.1 Model formulation

We consider three infection states for human population and categorize them into five compartments: susceptible (*S*_*h*_), asymptomatic infection (*A*_*h*_), acute infection (*I*_*ac*_), chronic infection (*I*_*ch*_), and recovered (*R*_*h*_). The total human population size is denoted by *T*_*h*_. This population experiences a growth rate *B*_*h*_ and a natural mortality rate *ϵ*_*h*_. Owing to the low fatality rate of Chikungunya, additional mortality from infection is not considered in this model. The per-bite transmission risk from mosquito to human is *ρ*_1_. It is assumed that infected individuals with a proportion *p* exhibit symptoms and progress to the acute stage, while the remainder become asymptomatic infected individuals. Asymptomatic individuals recover after a certain period, with a recovery rate of *η*_*h*_. In addition to recovering at rate *θ*_*h*_, a fraction of individuals in the acute stage advance into the chronic stage at rate *σ*_*h*_, and subsequently recover at rate *δ*_*h*_. All recovered individuals acquire permanent immunity and are not susceptible to reinfection.

We classify the mosquito population into two compartments: susceptible (*S*_*v*_) and infected (*I*_*v*_), and the total population is denoted by *T*_*v*_. *B*_*v*_ and *ϵ*_*v*_ are defined as the birth and mortality rates for the mosquito population, respectively. Since chronic carriers do not carry a sufficient viral load to infect mosquitoes, susceptible mosquitoes typically acquire infection through biting either asymptomatic or acutely infected human hosts. The infection rates from acutely infected and asymptomatic individuals to mosquitoes are given by *ρ*_2_ and *κρ*_2_, respectively.

Fig. 2.1 illustrates the transmission dynamics of Chikungunya fever. All parameters take on non-negative values. Table 2.1 summarizes the complete parameters and their biological interpretations. We can formulate the following ordinary differential equation (ODE) system:

(2.1) $$\left\{\begin{array}{l} \frac{dS_{h}}{dt}=B_{h}-\frac{\beta \rho_{1}I_{v}S_{h}}{T_{h}}-\epsilon_{h}S_{h},\;\\ \frac{dA_{h}}{dt}=\frac{\beta \rho_{1}\left(1-p\right)I_{v}S_{h}}{T_{h}}-\eta_{h}A_{h}-\epsilon_{h}A_{h},\;\\ \frac{dI_{ac}}{dt}=\frac{\beta \rho_{1}pI_{v}S_{h}}{T_{h}}-\sigma_{h}I_{ac}-\theta_{h}I_{ac}-\epsilon_{h}I_{ac},\;\\ \frac{dI_{ch}}{dt}=\sigma_{h}I_{ac}-\delta_{h}I_{ch}-\epsilon_{h}I_{ch},\;\\ \frac{dR_{h}}{dt}=\eta_{h}A_{h}+\theta_{h}I_{ac}+\delta_{h}I_{ch}-\epsilon_{h}R_{h},\;\\ \frac{dS_{v}}{dt}=B_{v}-\frac{\beta \rho_{2}\left(I_{ac}+\kappa A_{h}\right)S_{v}}{T_{h}}-\epsilon_{v}S_{v},\;\\ \frac{dI_{v}}{dt}=\frac{\beta \rho_{2}\left(I_{ac}+\kappa A_{h}\right)S_{v}}{T_{h}}-\epsilon_{v}I_{v}.\; \end{array}\right)$$

**Fig. 1:**
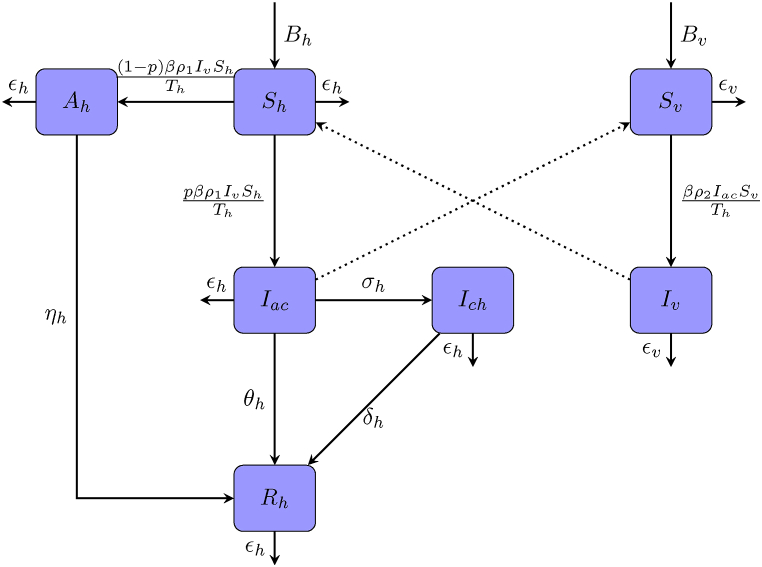
Key components and process of Chikungunya virus transmission.

**Table 2.1:** Summary of model parameters.

Symbol	Biological Meanings
*B*_*h*_	Susceptible individuals entry rate
*B*_*v*_	Susceptible mosquitoes entry rate
*β*	Mosquito biting rate
*ρ*_1_	Mosquito-to-human infection probability
*ρ*_2_	Human-to-mosquito infection probability
*η*_*h*_	Recovery rate for asymptomatic individuals
*p*	Proportion of cases that become symptomatic
*θ*_*h*_	Acute infected individuals recovery rate
*σ*_*h*_	Disease progression rate from acute to chronic phase
*δ*_*h*_	Chronic infected individuals recovery rate
*κ*	The ratio of the transmission of asymptomatic infections to that of acute infections
*ϵ*_*h*_	Human mortality rate
*ϵ*_*v*_	Mosquito mortality rate

Non-negative initial conditions are assumed for all state variables: *S*_*h*_(0) ≥ 0, *A*_*h*_(0) ≥ 0, *I*_*ac*_(0) ≥ 0, *I*_*ch*_(0) ≥ 0, *R*_*h*_(0) ≥ 0, *S*_*v*_(0) ≥ 0, *I*_*v*_(0) ≥ 0. The human and mosquito populations satisfy *T*_*h*_ = *S*_*h*_ + *A*_*h*_ + *I*_*ac*_ + *I*_*ch*_ + *R*_*h*_, and *T*_*v*_ = *S*_*v*_ + *I*_*v*_.

### 2.2 Model analysis

According to the model assumptions, we have: $\frac{dT_{h}}{dt}=B_{h}-\epsilon_{h}T_{h}$, $\frac{dT_{v}}{dt}=B_{v}-\epsilon_{h}T_{v}$. It is straightforward to show that: $\lim_{t\to \infty }T_{h}=\frac{B_{h}}{\epsilon_{h}}=T_{h}^{0}$, $\lim_{t\to \infty }T_{v}=\frac{B_{v}}{\epsilon_{v}}=T_{v}^{0}$.

Hence, the stability properties of original model is equivalent to that of the limiting system:

(2.2) $$\left\{\begin{array}{l} \frac{dS_{h}}{dt}=B_{h}-\frac{\beta \rho_{1}I_{v}S_{h}}{T_{h}^{0}}-\epsilon_{h}S_{h},\;\\ \frac{dA_{h}}{dt}=\frac{\beta \rho_{1}\left(1-p\right)I_{v}S_{h}}{T_{h}^{0}}-\eta_{h}A_{h}-\epsilon_{h}A_{h},\;\\ \frac{dI_{ac}}{dt}=\frac{\beta \rho_{1}pI_{v}S_{h}}{T_{h}^{0}}-\sigma_{h}I_{ac}-\theta_{h}I_{ac}-\epsilon_{h}I_{ac},\;\\ \frac{dI_{ch}}{dt}=\sigma_{h}I_{ac}-\delta_{h}I_{ch}-\epsilon_{h}I_{ch},\;\\ \frac{dR_{h}}{dt}=\eta_{h}A_{h}+\theta_{h}I_{ac}+\delta_{h}I_{ch}-\epsilon_{h}R_{h},\;\\ \frac{dS_{v}}{dt}=B_{v}-\frac{\beta \rho_{2}\left(I_{ac}+\kappa A_{h}\right)S_{v}}{T_{h}^{0}}-\epsilon_{v}S_{v},\;\\ \frac{dI_{v}}{dt}=\frac{\beta \rho_{2}\left(I_{ac}+\kappa A_{h}\right)S_{v}}{T_{h}^{0}}-\epsilon_{v}I_{v}.\; \end{array}\right)$$

As the variables *I*_*ch*_ and *R*_*h*_ are absent from other equations of the system, the subsequent analysis is restricted to the reduced system corresponding to *S*_*h*_, *A*_*h*_, *I*_*ac*_, *S*_*v*_, *I*_*v*_:

(2.3) $$\left\{\begin{array}{l} \frac{dS_{h}}{dt}=B_{h}-\frac{\beta \rho_{1}I_{v}S_{h}}{T_{h}^{0}}-\epsilon_{h}S_{h},\;\\ \frac{dS_{v}}{dt}=B_{v}-\frac{\beta \rho_{2}\left(I_{ac}+\kappa A_{h}\right)S_{v}}{T_{h}^{0}}-\epsilon_{v}S_{v},\;\\ \frac{dA_{h}}{dt}=\frac{\beta \rho_{1}\left(1-p\right)I_{v}S_{h}}{T_{h}^{0}}-\eta_{h}A_{h}-\epsilon_{h}A_{h},\;\\ \frac{dI_{ac}}{dt}=\frac{\beta \rho_{1}pI_{v}S_{h}}{T_{h}^{0}}-\sigma_{h}I_{ac}-\theta_{h}I_{ac}-\epsilon_{h}I_{ac},\;\\ \frac{dI_{v}}{dt}=\frac{\beta \rho_{2}\left(I_{ac}+\kappa A_{h}\right)S_{v}}{T_{h}^{0}}-\epsilon_{v}I_{v}.\; \end{array}\right)$$

Let $\beta_{1}=\frac{\beta \rho_{1}\epsilon_{h}}{B_{h}}$, $\beta_{2}=\frac{\beta \rho_{2}\epsilon_{h}}{B_{h}}$, $\beta_{3}=\frac{\kappa \beta \rho_{2}\epsilon_{h}}{B_{h}}$, *ϵ*_*a*_ = *ϵ*_*h*_ + *η*, *ϵ*_*ac*_ = *ϵ*_*h*_ + *σ*_*h*_ +*θ*_*h*_, *μ* = *ϵ*_*h*_, *d* = *ϵ*_*v*_, *d*_1_ = *ϵ*_*ac*_, *d*_2_ = *ϵ*_*a*_, *B*_1_ = *B*_*h*_, *B*_2_ = *B*_*v*_, *q* = 1 − *p*.

And We define the state variables as *x* = *S*_*h*_, *y* = *S*_*v*_, *u* = *I*_*ac*_, *v* = *A*_*h*_, *z* = *I*_*v*_.

Then the reduced system (2.3) takes the form:

(2.4) $$\left\{\begin{array}{l} \frac{dx}{dt}=B_{1}-\beta_{1}xz-\mu x,\;\\ \frac{dy}{dt}=B_{2}-\left(\beta_{2}u+\beta_{3}v\right)y-dy,\;\\ \frac{du}{dt}=p\beta_{1}xz-d_{1}u,\;\\ \frac{dv}{dt}=q\beta_{1}xz-d_{2}v,\;\\ \frac{dz}{dt}=\left(\beta_{2}u+\beta_{3}v\right)y-dz.\; \end{array}\right)$$

The system's equilibrium *E*∗ = (*x*∗, *y*∗, *u*∗, *v*∗, *z*∗) satisfies

$$\begin{array}{ll} 0 & =B_{1}-\beta_{1}x^{*}z^{*}-\mu x^{*},\\ 0 & =B_{2}-\left(\beta_{2}u^{*}+\beta_{3}v^{*}\right)y-dy,\\ 0 & =p\beta_{1}x^{*}z^{*}-d_{1}u^{*},\\ 0 & =q\beta_{1}x^{*}z^{*}-d_{2}v^{*},\\ 0 & =\left(\beta_{2}u^{*}+\beta_{3}v^{*}\right)y^{*}-dz^{*}. \end{array}$$

Solving these equations yields: $x^{*}=\frac{B_{1}}{\mu +\beta_{1}z^{*}}$, $y^{*}=\frac{B_{2}}{d}-z^{*}$, $u^{*}=\frac{pB_{1}\beta_{1}z^{*}}{d_{1}\left(\mu +\beta_{1}z^{*}\right)}$, $v^{*}=\frac{qB_{1}\beta_{1}z^{*}}{d_{2}\left(\mu +\beta_{1}z^{*}\right)}$, $\left(\beta_{2}\frac{pB_{1}\beta_{1}z^{*}}{d_{1}\left(\mu +\beta_{1}z^{*}\right)}+\beta_{3}\frac{qB_{1}\beta_{1}z^{*}}{d_{2}\left(\mu +\beta_{1}z^{*}\right)}\right)\left(\frac{B_{2}}{d}-z^{*}\right)=dz^{*}$.

Thus, the system has two equilibria:

• The disease-free equilibrium (*z*∗ = 0): $E_{0}=\left(\frac{B_{1}}{\mu },\frac{B_{2}}{d},0,0,0\right)=\left(\frac{B_{h}}{\epsilon_{h}},\frac{B_{v}}{\epsilon_{v}},0,0,0\right)$.

Computation of the basic reproduction number, based on the next-generation matrix (Van Den Driessche & Watmough, 2002), leads to the following expression:

$$R_{0}=\sqrt{\frac{\beta^{2}\rho_{1}\rho_{2}B_{v}\epsilon_{h}}{B_{h}\epsilon_{v}^{2}}\left(\frac{p}{\sigma_{h}+\theta_{h}+\epsilon_{h}}+\frac{\kappa \left(1-p\right)}{\eta_{h}+\epsilon_{h}}\right)}.$$

Let $R_{0}^{2}=pR_{1}+qR_{2}$, where $R_{1}=\frac{\beta_{1}\beta_{2}B_{1}B_{2}}{\mu d_{1}d^{2}}=\frac{\beta_{1}\beta_{2}x_{0}y_{0}}{dd_{1}}$, $R_{2}=\frac{\beta_{1}\beta_{3}B_{1}B_{2}}{\mu d_{2}d^{2}}=\frac{\beta_{1}\beta_{3}x_{0}y_{0}}{dd_{2}}$.

• The endemic equilibrium (*z*∗ ≠ 0): $E^{*}=\left(S_{h}^{*},S_{v}^{*},A_{h}^{*},I_{ac}^{*},I_{v}^{*}\right)$, where

$$\begin{array}{ll} & S_{h}^{*}=\frac{B_{h}T_{h}^{0}}{T_{h}^{0}\epsilon_{h}+\beta \rho_{1}I_{v}^{*}},\;A_{h}^{*}=\frac{\left(1-p\right)\beta \rho_{1}B_{h}I_{v}^{*}}{\left(\eta_{h}+\epsilon_{h}\right)\left(T_{h}^{0}\epsilon_{h}+\beta \rho_{1}I_{v}^{*}\right)},\\ & I_{ac}^{*}=\frac{p\beta \rho_{1}B_{h}I_{v}^{*}}{\left(\sigma_{h}+\theta_{h}+\epsilon_{h}\right)\left(T_{h}^{0}\epsilon_{h}+\beta \rho_{1}I_{v}^{*}\right)},\;S_{v}^{*}=\frac{B_{v}}{\epsilon_{v}}-I_{v}^{*},\\ & I_{v}^{*}=\frac{{\left(T_{h}^{0}\right)}^{2}\epsilon_{v}^{2}\epsilon_{h}}{\beta^{2}\rho_{1}\rho_{2}B_{h}\epsilon_{v}\left(\frac{p}{\sigma_{h}+\theta_{h}+\epsilon_{h}}+\frac{\kappa \left(1-p\right)}{\eta_{h}+\epsilon_{h}}\right)+\beta \rho_{1}T_{h}^{0}\epsilon_{v}^{2}}\left(R_{0}^{2}-1\right) \end{array}$$

Theorem 2.1 *When* *R*_0_ < 1*, the disease-free equilibrium* *E*_0_ *of system* (2.4) *is globally asymptotically stable.*

Proof. Consider the Jacobian matrix derived from the linearization of system (2.4) at *E*_0_:

$$J\left(E_{0}\right)=\left(\begin{array}{lllll} -\mu & 0 & 0 & 0 & -\beta_{1}x_{0}\\ 0 & -d & -\beta_{2}y_{0} & -\beta_{3}y_{0} & 0\\ 0 & 0 & -d_{1} & 0 & p\beta_{1}x_{0}\\ 0 & 0 & 0 & -d_{2} & q\beta_{1}x_{0}\\ 0 & 0 & \beta_{2}y_{0} & \beta_{3}y_{0} & -d \end{array}\right).$$

Then, $det\left(\lambda E-J\left(E_{0}\right)\right)=\left(\lambda +\mu \right)\left(\lambda +d\right)D\left(\lambda \right)=0$, where, $D\left(\lambda \right)=\lambda^{3}+\left(d+d_{1}+d_{2}\right)\lambda^{2}+\lambda \left(dd_{1}\left(1-pR_{1}\right)+dd_{2}\left(1-qR_{2}\right)+d_{1}d_{2}\right)+dd_{1}d_{2}\left(1-R_{0}^{2}\right)$. The condition *R*_0_ < 1 ensures that all roots of *D*(*λ*) = 0 possess negative real parts, thereby ensuring the local asymptotic stability of *E*_0_.

Let *P*(*x*) = *x* − 1 − ln *x*. Consider the following Lyapunov function:

$$L_{1}\left(t\right)=x_{0}P\left(\frac{x}{x_{0}}\right)+u+v+K_{1}\left(y_{0}P\left(\frac{y}{y_{0}}\right)+z\right),$$

where $K_{1}=\frac{\beta_{1}x_{0}}{d}$, and

$$\begin{array}{ll} {\left(x_{0}P\left(\frac{x}{x_{0}}\right)+u+v\right)}^{\prime } & =\left(1-\frac{x_{0}}{x}\right)\left(B_{1}-\beta_{1}xz-\mu x\right)+\beta_{1}xz-d_{1}u-d_{2}v\\ & =\mu x_{0}\left(1-\frac{x_{0}}{x}\right)\left(1-\frac{x}{x_{0}}\right)+\beta_{1}x_{0}z-d_{1}u-d_{2}v, \end{array}$$

$$\begin{array}{ll} {\left(y_{0}P\left(\frac{y}{y_{0}}\right)+z\right)}^{\prime } & =\left(1-\frac{y_{0}}{y}\right)\left(B_{2}-\beta_{2}uy-\beta_{3}vy-dy\right)+\beta_{2}uy+\beta_{3}vy-dz\\ & =dy_{0}\left(1-\frac{y_{0}}{y}\right)\left(1-\frac{y}{y_{0}}\right)+\beta_{2}uy_{0}+\beta_{3}vy_{0}-dz. \end{array}$$

It follows that $\frac{dL_{1}}{dt}=\mu x_{0}\left(2-\frac{x_{0}}{x}-\frac{x}{x_{0}}\right)+K_{1}dy_{0}\left(2-\frac{y_{0}}{y}-\frac{y}{y_{0}}\right)+d_{1}u\left(R_{1}-1\right)+d_{2}v\left(R_{2}-1\right)$.

We obtain $\frac{dL_{1}}{dt}\leq 0$, and $\frac{dL_{1}}{dt}=0$, if and only if *x* = *x*_0_, *y* = *y*_0_, *u* = *v* = 0. The global asymptotic stability of the disease-free equilibrium is established via LaSalle's invariance principle (LaSalle, 1976).

Theorem 2.2 *When* *R*_0_ > 1*, the endemic equilibrium* *E*∗ *is globally asymptotically stable.*

Proof. Consider the Jacobian matrix derived from the linearization of system (2.4) at *E*∗:

$$J\left(E^{*}\right)=\left(\begin{array}{lllll} -\mu -\beta_{1}z^{*} & 0 & 0 & 0 & -\beta_{1}x^{*}\\ 0 & -d-\left(\beta_{2}u^{*}+\beta_{3}v^{*}\right) & -\beta_{2}y^{*} & -\beta_{3}y^{*} & 0\\ p\beta_{1}z^{*} & 0 & -d_{1} & 0 & p\beta_{1}x^{*}\\ q\beta_{1}z^{*} & 0 & 0 & -d_{2} & q\beta_{1}x^{*}\\ 0 & \beta_{2}u^{*}+\beta_{3}v^{*} & \beta_{2}y^{*} & \beta_{3}y^{*} & -d \end{array}\right).$$

We denote *k* = *β*_2_*u*∗ + *β*_3_*v*∗. The characteristic equation is $det\left(\lambda E-J\left(E^{*}\right)\right)=\left(\lambda +d\right)\hat{D}\left(\lambda \right)=0$, where, $\hat{D}\left(\lambda \right)=\left(\lambda +\mu +\beta_{1}z^{*}\right)\left(\lambda +d_{2}\right)\left(\lambda +d+k\right)\left(\lambda +d_{1}\right)-\left(\lambda +\mu \right)\left[\left(\lambda +d_{1}\right)q\beta_{1}x^{*}\beta_{3}y^{*}+\left(\lambda +d_{2}\right)p\beta_{1}x^{*}\beta_{2}y^{*}\right]$.

From *pβ*_1_*x*∗*z*∗ = *d*_1_*u*∗, *qβ*_1_*x*∗*z*∗ = *d*_2_*v*∗, *β*_2_*u*∗*y*∗ + *β*_3_*v*∗*y*∗ = *dz*∗, $d\left(d_{1}+d_{2}\right)=\frac{d\beta_{2}u^{*}y^{*}+\beta_{3}v^{*}y^{*}}{z^{*}}\left(d_{1}+d_{2}\right)=q\beta_{1}x^{*}\beta_{3}y^{*}+p\beta_{1}x^{*}\beta_{2}y^{*}+\frac{pd_{2}\beta_{1}\beta_{2}x^{*}y^{*}}{d_{1}}+\frac{qd_{1}\beta_{1}\beta_{3}x^{*}y^{*}}{d_{2}}$, we can obtain

$$\begin{array}{ll} \hat{D}\left(\lambda \right) & =\left(\lambda +\mu +\beta_{1}z^{*}\right)\left(\lambda +d+k\right)\left(\lambda +d_{1}\right)(\lambda +d_{2})-\left(\lambda +\mu \right)\left(d\left(d_{1}+d_{2}\right)\lambda -\left(\frac{pd_{2}\beta_{1}\beta_{2}x^{*}y^{*}}{d_{1}}+\frac{qd_{1}\beta_{1}\beta_{3}x^{*}y^{*}}{d_{2}}\right)\lambda +dd_{1}d_{2}\right)\\ & =\lambda^{4}+c_{1}\lambda^{3}+c_{2}\lambda^{2}+c_{3}\lambda +c_{4}, \end{array}$$

where $A=\frac{pd_{2}\beta_{1}\beta_{2}x^{*}y^{*}}{d_{1}}+\frac{qd_{1}\beta_{1}\beta_{3}x^{*}y^{*}}{d_{2}}<d\left(d_{1}+d_{2}\right)$, $\hat{A}=A+d_{1}d_{2}$. We have

$$\begin{array}{ll} c_{1} & =\mu +\beta_{1}z^{*}+d+k+d_{1}+d_{2}>0,\\ c_{2} & =\left(\mu +\beta_{1}z^{*}\right)\left(d+k+d_{1}+d_{2}\right)+k\left(d_{1}+d_{2}\right)+\hat{A}>0,\\ c_{3} & =\beta_{1}z^{*}\left(dd_{1}+dd_{2}+d_{1}d_{2}+k\left(d_{1}+d_{2}\right)\right)+\mu k\left(d_{1}+d_{2}\right)+kd_{1}d_{2}+\mu \hat{A}>0,\\ c_{4} & =\beta_{1}z^{*}d_{1}d_{2}\left(d+k\right)+\mu kd_{1}d_{2}>0. \end{array}$$

Therefore, we obtain:

$$\begin{array}{ll} \Delta_{2} & =c_{1}c_{2}-c_{3}\\ & ={\left(\beta_{1}z^{*}\right)}^{2}(d+k+d_{1}+d_{2})+\beta_{1}z^{*}\left(d_{2}^{2}+d_{1}^{2}+{\left(d+k\right)}^{2}+dd_{1}+dd_{2}+d_{1}d_{2}+\hat{A}+2\mu \left(d+k+d_{1}+d_{2}\right)+2k\left(d_{1}+d_{2}\right)\right)\\ & \;+\mu {\left(d_{2}+d_{1}+d+k\right)}^{2}+\left(d_{2}+d_{1}+d+k\right)\hat{A}+\mu^{2}(d_{2}+d_{1}+d+k)+k\left[d_{1}^{2}+d_{2}^{2}+d_{1}d_{2}+\left(d+k\right)\left(d_{1}+d_{2}\right)\right],\\ \Delta_{3} & =c_{3}\Delta_{2}-c_{1}^{2}c_{4}={\left(\beta_{1}z^{*}\right)}^{3}A_{3}+{\left(\beta_{1}z^{*}\right)}^{2}A_{2}+\beta_{1}z^{*}A_{1}+A_{0}, \end{array}$$

where

$$\begin{array}{ll} A_{0} & =\left[\mu {\left(d+k+d_{1}+d_{2}\right)}^{2}+\left(d+k+d_{1}+d_{2}\right)\left(\hat{A}+\mu^{2}\right)+A_{4}\right]\left(\mu \hat{A}+\mu k\left(d_{1}+d_{2}\right)+kd_{1}d_{2}\right)-\mu kd_{1}d_{2}{\left(\mu +d+k+d_{1}+d_{2}\right)}^{2}>0,\\ A_{1} & =\left[\mu {\left(d+k+d_{1}+d_{2}\right)}^{2}+\left(d+k+d_{1}+d_{2}\right)\left(\hat{A}+\mu^{2}\right)+A_{4}\right][d_{1}d_{2}+\left(d+k\right)\left(d_{1}+d_{2}\right)]+\left[\mu k\left(d_{1}+d_{2}\right)+kd_{1}d_{2}+\mu \hat{A}\right]A_{5}\\ & \;-2\mu kd_{1}d_{2}\left(\mu +d+k+d_{1}+d_{2}\right)-d_{1}d_{2}\left(d+k\right){\left(\mu +d+k+d_{1}+d_{2}\right)}^{2}>0,\\ A_{2} & =\left[dd_{1}+dd_{2}+d_{1}d_{2}+k\left(d_{1}+d_{2}\right)\right]A_{5}+\left(d+k+d_{1}+d_{2}\right)\left[\mu k\left(d_{1}+d_{2}\right)+kd_{1}d_{2}+\mu \hat{A}\right]\\ & \;-\mu kd_{1}d_{2}-2d_{1}d_{2}\left(d+k\right)\left(\mu +d+k+d_{1}+d_{2}\right)>0,\\ A_{3} & =\left(d+k+d_{1}+d_{2}\right)\left[dd_{1}+dd_{2}+d_{1}d_{2}+k\left(d_{1}+d_{2}\right)\right]-\left(d+k\right)d_{1}d_{2}>0,\\ A_{4} & =k\left(d_{1}^{2}+d_{2}^{2}+d_{1}d_{2}+\left(d+k\right)\left(d_{1}+d_{2}\right)\right),\\ A_{5} & =d_{2}^{2}+d_{1}^{2}+{\left(d+k\right)}^{2}+d_{1}d_{2}+\hat{A}+2\mu \left(d+k+d_{1}+d_{2}\right)+\left(2k+d\right)\left(d_{1}+d_{2}\right) \end{array}$$

An application of the Hurwitz criterion confirms that all roots of $\hat{D}\left(\lambda \right)=0$ possess negative real parts, implying that the same holds for all roots of $det\left(\lambda E-J\left(E^{*}\right)\right)=0$. This satisfies the criteria for the local asymptotic stability of *E*∗.

Next, define a Lyapunov function as follows:

$$L_{2}\left(t\right)=\left(K_{2}p+K_{3}q\right)x^{*}P\left(\frac{x}{x^{*}}\right)+K_{2}u^{*}P\left(\frac{u}{u^{*}}\right)+K_{3}v^{*}P\left(\frac{v}{v^{*}}\right)+y^{*}P\left(\frac{y}{y^{*}}\right)+z^{*}P\left(\frac{z}{z^{*}}\right),$$

where $K_{2}=\frac{\beta_{2}u^{*}y^{*}}{p\beta_{1}x^{*}z^{*}}$, $K_{3}=\frac{\beta_{3}v^{*}y^{*}}{q\beta_{1}x^{*}z^{*}}$. Then

$$\begin{array}{ll} & {\left[px^{*}P\left(\frac{x}{x^{*}}\right)+u^{*}P\left(\frac{u}{u^{*}}\right)\right]}^{'}=p\left(1-\frac{x^{*}}{x}\right)\left(B_{1}-\beta_{1}xz-\mu x\right)+\left(1-\frac{u^{*}}{u}\right)\left(p\beta_{1}xz-p\beta_{1}x^{*}z^{*}\frac{u}{u^{*}}\right)\\ & \;\;\;\;\;\;\;\;\;\;\;\;\;\;\;\;\;\;\;\;\;\;\;\;\;\;\;\;\;\;\;\;\;\;\;\;\;\;\;\;\;\;\;\;=p\mu x^{*}\left(1-\frac{x^{*}}{x}\right)\left(1-\frac{x}{x^{*}}\right)+p\beta_{1}x^{*}z^{*}\left(\left(1-\frac{xz}{x^{*}z^{*}}\right)\left(1-\frac{x^{*}}{x}\right)+\left(1-\frac{u^{*}}{u}\right)\left(\frac{xz}{x^{*}z^{*}}-\frac{u}{u^{*}}\right)\right)\\ & \;\;\;\;\;\;\;\;\;\;\;\;\;\;\;\;\;\;\;\;\;\;\;\;\;\;\;\;\;\;\;\;\;\;\;\;\;\;\;\;\;\;\;\;=p\mu x^{*}\left(2-\frac{x^{*}}{x}-\frac{x}{x^{*}}\right)+p\beta_{1}x^{*}z^{*}\left(2+\frac{z}{z^{*}}-\frac{x^{*}}{x}-\frac{xzu^{*}}{x^{*}z^{*}u}-\frac{u}{u^{*}}\right), \end{array}$$

$$\begin{array}{ll} & {\left[qx^{*}P\left(\frac{x}{x^{*}}\right)+v^{*}P\left(\frac{v}{v^{*}}\right)\right]}^{'}=q\left(1-\frac{x^{*}}{x}\right)\left(B_{1}-\beta_{1}xz-\mu x\right)+\left(1-\frac{v^{*}}{v}\right)\left(q\beta_{1}xz-q\beta_{1}x^{*}z^{*}\frac{v}{v^{*}}\right)\\ & \;\;\;\;\;\;\;\;\;\;\;\;\;\;\;\;\;\;\;\;\;\;\;\;\;\;\;\;\;\;\;\;\;\;\;\;\;\;\;\;\;\;\;\;=q\mu x^{*}\left(1-\frac{x^{*}}{x}\right)\left(1-\frac{x}{x^{*}}\right)+q\beta_{1}x^{*}z^{*}\left(\left(1-\frac{xz}{x^{*}z^{*}}\right)\left(1-\frac{x^{*}}{x}\right)+\left(1-\frac{v^{*}}{v}\right)\left(\frac{xz}{x^{*}z^{*}}-\frac{v}{v^{*}}\right)\right)\\ & \;\;\;\;\;\;\;\;\;\;\;\;\;\;\;\;\;\;\;\;\;\;\;\;\;\;\;\;\;\;\;\;\;\;\;\;\;\;\;\;\;\;\;\;=q\mu x^{*}\left(2-\frac{x^{*}}{x}-\frac{x}{x^{*}}\right)+q\beta_{1}x^{*}z^{*}\left(2+\frac{z}{z^{*}}-\frac{x^{*}}{x}-\frac{xzv^{*}}{x^{*}z^{*}v}-\frac{v}{v^{*}}\right), \end{array}$$

$$\begin{array}{ll} & {\left[y^{*}P\left(\frac{y}{y^{*}}\right)+z^{*}P\left(\frac{z}{z^{*}}\right)\right]}^{'}=\left(1-\frac{y^{*}}{y}\right)\left(B_{2}-\beta_{2}uy-\beta_{3}vy-dy\right)+\left(1-\frac{u^{*}}{u}\right)\left(\beta_{2}uy+\beta_{3}vy-\left(\beta_{2}u^{*}y^{*}+\beta_{3}v^{*}y^{*}\right)\frac{z}{z^{*}}\right)\\ & \;\;\;\;\;\;\;\;\;\;\;\;\;\;\;\;\;\;\;\;\;\;\;\;\;\;\;\;\;\;\;\;\;\;\;\;\;\;\;\;\;\;=dy^{*}\left(1-\frac{y^{*}}{y}\right)\left(1-\frac{y}{y^{*}}\right)+\beta_{2}u^{*}y^{*}\left(\left(1-\frac{y^{*}}{y}\right)\left(1-\frac{uy}{u^{*}y^{*}}\right)+\left(1-\frac{z^{*}}{z}\right)\left(\frac{uy}{u^{*}y^{*}}-\frac{z}{z^{*}}\right)\right)\\ & \;\;\;\;\;\;\;\;\;\;\;\;\;\;\;\;\;\;\;\;\;\;\;\;\;\;\;\;\;\;\;\;\;\;\;\;\;\;\;\;\;\;\;\;\;+\beta_{3}v^{*}y^{*}\left(\left(1-\frac{y^{*}}{y}\right)\left(1-\frac{vy}{v^{*}y^{*}}\right)+\left(1-\frac{z^{*}}{z}\right)\left(\frac{vy}{v^{*}y^{*}}-\frac{z}{z^{*}}\right)\right)\\ & \;\;\;\;\;\;\;\;\;\;\;\;\;\;\;\;\;\;\;\;\;\;\;\;\;\;\;\;\;\;\;\;\;\;\;\;\;\;\;\;\;\;=dy^{*}\left(2-\frac{y^{*}}{y}-\frac{y}{y^{*}}\right)+\beta_{2}u^{*}y^{*}\left(2-\frac{z}{z^{*}}-\frac{y^{*}}{y}-\frac{z^{*}yu}{zy^{*}u^{*}}+\frac{u}{u^{*}}\right)\;+\beta_{3}v^{*}y^{*}\left(2-\frac{z}{z^{*}}-\frac{y^{*}}{y}-\frac{z^{*}yv}{zy^{*}v^{*}}+\frac{v}{v^{*}}\right). \end{array}$$

Therefore,

$$\begin{array}{ll} \frac{dL_{2}}{dt} & =\left(K_{2}+K_{3}\right)\mu x^{*}\left(1-\frac{x^{*}}{x}\right)\left(1-\frac{x}{x^{*}}\right)+dy^{*}\left(1-\frac{y^{*}}{y}\right)\left(1-\frac{y}{y^{*}}\right)+\beta_{2}u^{*}y^{*}\left(4-\frac{x^{*}}{x}-\frac{xzu^{*}}{x^{*}z^{*}u}-\frac{y^{*}}{y}-\frac{z^{*}yu}{zy^{*}u^{*}}\right)\\ & \;+\beta_{3}v^{*}y^{*}\left(4-\frac{x^{*}}{x}-\frac{xzv^{*}}{x^{*}z^{*}v}-\frac{y^{*}}{y}-\frac{z^{*}yv}{zy^{*}v^{*}}\right). \end{array}$$

This shows that $\frac{dL_{2}}{dt}\leq 0$ and $\frac{dL_{2}}{dt}=0$ if and only if *x* = *x*∗, *y* = *y*∗, *u* = *u*∗, *v* = *v*∗, *z* = *z*∗. An application of LaSalle's invariance theorem (LaSalle, 1976) demonstrates the endemic equilibrium is globally asymptotically stable.

## 3 Parameter estimation and stability simulation

### 3.1 Fixed parameters and data

The recent Chikungunya fever outbreak in Foshan City remained primarily concentrated in the Shunde District. Therefore, we collect the daily reported number of new Chikungunya cases in Shunde District from July 19 to August 11, 2025 from Foshan Municipal Health Bureau, as shown in Table 3.2. According to information provided by the Foshan Municipal Bureau of Statistics, the total population of Shunde District in 2025 is *T*_*h*_ = 3268600, with a mean life expectancy of 83.6 years. From this, the mortality rate can be estimated as $\epsilon_{h}=\frac{1}{83.6\times 365}\approx 0.000033$. Under the assumption of equal birth and death rates, we have *B*_*h*_ = 3268600 × *ϵ*_*h*_ ≈ 107. Although asymptomatic infected individuals exhibit viremia, their viral load is significantly lower than that of symptomatic infected individuals (Appassakij et al., 2013). Consequently, their efficiency in vector-borne transmission to mosquitoes is reduced and then we estimate that *κ* = 0.3. The duration of acute infection is typically around 7 days (Rama et al., 2024), hence the removal rate from the acute infection compartment is estimated to be 0.143. Given that approximately 30–40 % of patients may progress to become chronically infected, we set *θ*_*h*_ = 0.086 and *σ*_*h*_ = 0.057. Accordingly, we assign the fixed biological parameters based on literature sources, including key epidemiological parameters such as total population size (*T*_*h*_), population growth rate (*B*_*h*_), and clinical progression rate (*σ*_*h*_, *θ*_*h*_, *η*_*h*_, *δ*_*h*_). Table 3.3 lists the specific parameter values used in the model.

**Table 3.2:** New daily cases in Shunde district.

Jul 19	Jul 20	Jul 21	Jul 22	Jul 23	Jul 24	Jul 25	Jul 26
629	368	313	463	383	310	273	310

Jul 27	Jul 28	Jul 29	Jul 30	Jul 31	Aug 1	Aug 2	Aug 3
323	357	408	362	312	258	192	158

Aug 4	Aug 5	Aug 6	Aug 7	Aug 8	Aug 9	Aug 10	Aug 11
135	122	115	111	102	98	77	68

**Table 3.3:** Fixed parameters and their values.

Symbol	Values	Reference
*η*_*h*_	0.167	Feng et al., 2019Feng et al. (2019)
*p*	0.86	Feng et al., 2019Feng et al. (2019)
*σ*_*h*_	0.057	See text
*θ*_*h*_	0.086	See text
*δ*_*h*_	0.003	González-Parra et al., 2019González-Parra et al. (2019)
*κ*	0.3	See text
*ϵ*_*v*_	0.07	González-Parra et al., 2019González-Parra et al. (2019)
*T*_*h*_	3268600	See text
*B*_*h*_	107	See text
*ϵ*_*h*_	0.000033	See text

### 3.2 Parameter fitting

This study employs a parameter estimation framework based on the least squares method to fit the daily new case data within the MATLAB environment. The framework is used to estimate three key parameters—the mosquito biting rate (*β*), the human infection probability (*ρ*_1_), and the mosquito infection probability (*ρ*_2_), along with the total mosquito population (*T*_*v*_) and its initial prevalence of infection (*I*_*v*_(0)). To comprehensively evaluate the model's goodness of fit and predictive capability, the collected daily case data are divided into two segments: the first 17 days for parameter estimation, and the subsequent 7 days for validating the model's predictive performance.

The original epidemic data may be subject to reporting delays and random fluctuations. To improve the quality of model fitting, this study applies the Weighted Moving Average (WMA) method for data smoothing. Specifically, a 5-day sliding window is employed, assigning higher weight to recent data to reduce random noise while preserving essential data characteristics. The weight distribution is generated using an exponential function with decay parameter *λ* = 1.7. The weight vector *ω* = [*ω*_1_, *ω*_2_, *ω*_3_, *ω*_4_, *ω*_5_] is computed as follows:

$$\omega_{i}=\frac{e^{\lambda i}}{\sum_{j=1}^{5}e^{\lambda i}},i=1,2,3,4,5.$$

For the first five data points (*t* ≤ 5), a simple arithmetic mean is applied. For *t* > 5, the smoothed value $\tilde{C}\left(t\right)$ is calculated as:

$$\tilde{C}\left(t\right)=\overset{5}{\underset{i=1}{\sum }}\omega_{i}C\left(t-5+i\right),$$

where *C*(*t*) denotes the observed value at time *t*.

As shown in Fig. 3.2, the comparison between raw and smoothed data demonstrates that the smoothed curve retains the overall trend of the outbreak development while significantly reducing short-term fluctuations and noise.

**Fig. 2:**
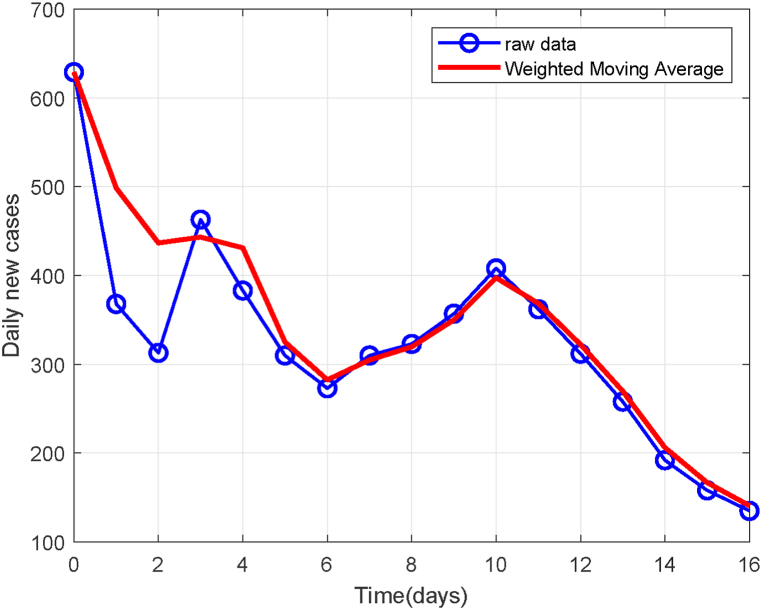
Comparison of data before and after smoothing processing.

Based on the characteristics of the early outbreak phase, initial values for the five epidemiological state in humans on July 19th are estimated as follows: susceptible individuals (*S*_*h*_), asymptomatic infected individuals (*A*_*h*_), symptomatic infected individuals in the acute stage (*I*_*ac*_), chronically infected individuals (*I*_*ch*_), and recovered individuals (*R*_*h*_). The assigned values are: *A*_*h*_ = 163, *I*_*ac*_ = 1000, *I*_*ch*_ = 10, *R*_*h*_ = 151, *S*_*h*_ = *T*_*h*_ − *A*_*h*_ − *I*_*ac*_ − *I*_*ch*_ − *R*_*h*_. Using the smoothed data, parameter optimization is performed via the nonlinear least squares method starting from day 1 (corresponding to July 1st). The lsqnonlin solver (based on Levenberg–Marquardt algorithm) is employed to minimize the residual sum of squares between simulated and actual values. The model yields a numerical solution for the daily new cases, denoted as $\hat{C}\left(t\right)$, computed by:

$$\hat{C}\left(t\right)=\frac{\beta \rho_{1}pI_{v}S_{h}}{T_{h}},$$

resulting in the estimated values $\hat{C}\left(0\right)$, $\hat{C}\left(1\right)$, …, $\hat{C}\left(16\right)$. The objective function is formulated as the sum of squared errors (SSE) across all predicted and observed data points:

$$SSE=\overset{16}{\underset{i=0}{\sum }}{\left(\hat{C}\left(i\right)-C\left(i\right)\right)}^{2}.$$

The coefficient of determination *R*^2^ is used to measure how closely the model's predictions aligned with the observations. It quantifies the proportion of variance explained by the model and is computed as:

$$R^{2}=1-\frac{SSE}{\sum_{i=0}^{16}\left(C\left(i\right)-\bar{C}\left(i\right)\right)},$$

where $\bar{C}$ denotes the mean of the observed daily case counts over the fitting period.

To ensure biologically plausible and numerically stable parameter estimation, search bounds are imposed on each parameter during optimization. The specific ranges provided in Table 3.4 are determined through preliminary fitting and practical rationale to enhance optimization efficiency by restricting the search to a realistic parameter space. We employ a bootstrap resampling method to quantify parameter estimation uncertainty by computing 95 % confidence intervals. Based on 1000 resamplings of the model residuals, the confidence intervals are defined by the 2.5th and 97.5th percentiles of the resulting empirical distribution.

**Table 3.4:** Parameters for estimation and their range.

Symbol	Value range	Symbol	Value range
*β*	[0.01,0.7]	*ρ*_1_	[0.01,0.7]
*ρ*_1_	[0.01,0.7]	*T*_*v*_	[1 × 10^6^,2 × 10^7^]
*I*_*v*_(0)	[1,1 × 10^5^]		

Fig. 3.3 presents the fitting results, with the estimated parameter values and goodness-of-fit metrics provided in Table 3.5. All parameters have plausible 95 % confidence intervals. The results suggest that the Chikungunya transmission model developed in this study achieves a moderate level of fitting accuracy (*R*^2^ = 0.76). The maximum residual is approximately 100 cases, suggesting that the model did not fully capture all variations in the epidemic time series. This is common in complex mosquito-borne disease systems, as transmission is influenced by numerous unobserved stochastic factors, such as human mobility and fluctuations in case reporting rates. Nevertheless, the model successfully captures the core dynamic trend of the outbreak and identified intrinsic driving forces of disease spread. The residuals are randomly distributed over time, and smaller residuals in later stages indicate that the model mechanism is relatively reliable during this period. Overall, the model demonstrates satisfactory fitting result, successfully quantifies key transmission parameters, and provides valuable mechanistic insights into the epidemic dynamics. Its limitations also offer clear directions for future model improvements.

**Fig. 3:**
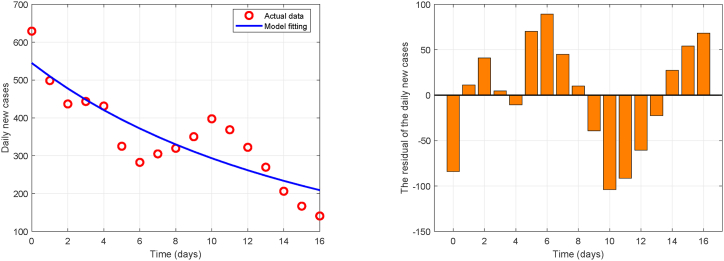
Presentation of fitting results.

**Table 3.5:** Model parameter estimates with 95 % confidence intervals.

Parameters	Value	95 % CI
*β*	0.2111	[0.0466,0.2411]
*ρ*_1_	0.1932	[0.1715,0.3193]
*ρ*_2_	0.1118	[0.0152,0.2232]
*T*_*v*_	6071859	[1.4562 × 10^6^,7.7513 × 10^6^]
*I*_*v*_(0)	15536	[1.3153 × 10^4^,4.5265 × 10^4^]
*R*^2^	0.76	

As shown in Fig. 3.4, although the actual observed values during the validation period (the last 7 days) mostly fall within the 95 % confidence interval of the model predictions, they consistently cluster near the lower boundary of the prediction interval. This non-random, systematic pattern suggests that while the model captures the overall declining trend of the outbreak, it may have systematically overestimated the intensity of viral transmission during the validation period. We hypothesize that this deviation likely reflects key differences in transmission dynamics between the model calibration period (the first 17 days) and the validation period. A plausible explanation is that around the beginning of the validation period, external mitigating factors begin to take significant effect. These include targeted mosquito control measures implemented after the peak transmission period, which reducing mosquito density, and increased public risk awareness leading to widespread individual protective behaviors (such as repellent use and reducing exposure). Together, these factors effectively suppress the actual transmission level, keeping it consistently lower than the model predictions based on the natural transmission dynamics observed in the earlier phase. Furthermore, according to actual reports, Foshan City activated an emergency response mechanism immediately after the outbreak was detected, focusing on three key interventions: environmental sanitation management, breeding site cleanup, and adult mosquito elimination. A targeted campaign for adult mosquito eradication was launched on July 31, aiming to comprehensively reduce mosquito populations. This real-world context aligns closely with our inferred causes. The systematic deviation of observed values below the predictions provides clear visual and quantitative evidence of the substantial effectiveness of the aforementioned interventions and behavioral changes in the population.

**Fig. 4:**
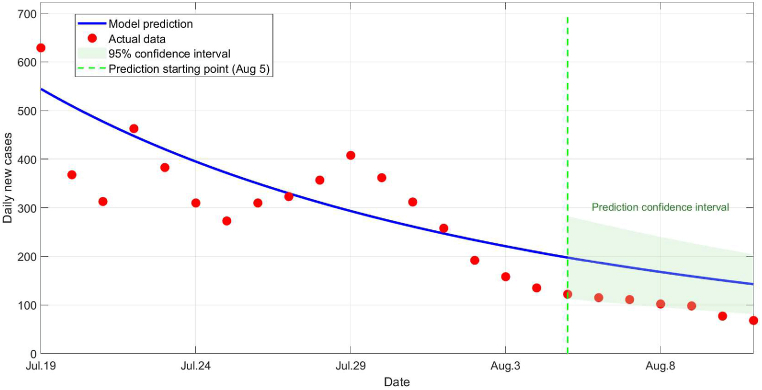
Comparison chart between model prediction and actual situation.

### 3.3 Stability simulation

This subsection presents numerical simulations that verify the stability of equilibrium points in system (2.1). We employ the ode45 solver in MATLAB to numerically solve this system of ordinary differential equations. The initial values are chosen as follows: *S*_*h*_(0) = 3267276, *A*_*h*_(0) = 163, *I*_*ac*_(0) = 1000, *I*_*ch*_(0) = 10, *S*_*v*_(0) = 6056359, *I*_*v*_(0) = 15536. For the given initial conditions and parameter set, we calculate *R*_0_ = 0.40 < 1. The stability of the equilibrium of system (2.1) can be observed in Fig. 3.5, which depicts the decay of both human and mosquito infections to zero and eventual stabilization. This result validates Theorem 2.1, indicating that disease elimination is the eventual outcome.

**Fig. 5:**
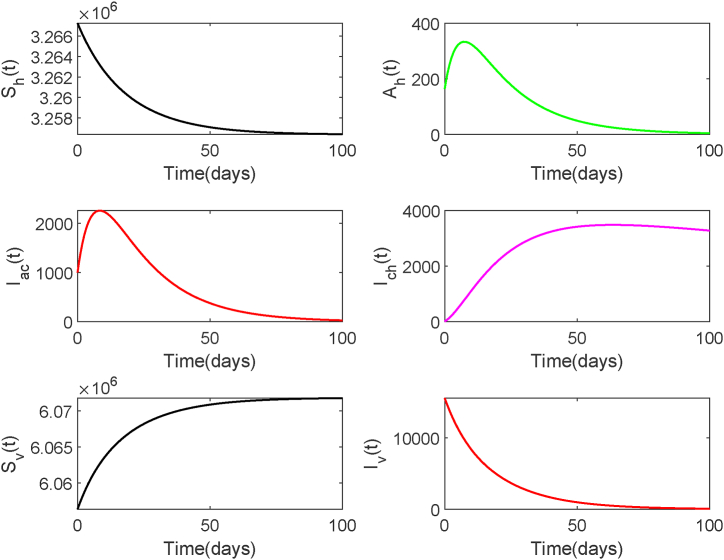
Solution Trajectories of system (2.1) when *R*_0_ < 1.

Next, we assume climatic conditions become favorable, leading to an increased mosquito reproduction rate and extended survival time. Accordingly, we set *ϵ*_*v*_ = 0.04, *B*_*v*_ = 2091904, while keeping all other parameters and initial values unchanged. Under this scenario, the basic reproduction number is *R*_0_ = 1.01 > 1. We observe from Fig. 3.6 that the equilibrium of system (2.1) is stable, which displays the numbers of asymptomatically infected individuals, acutely infected individuals, and infected mosquitoes stabilize at positive values, indicating disease persistence. This result provides validation for Theorem 2.2.

**Fig. 6:**
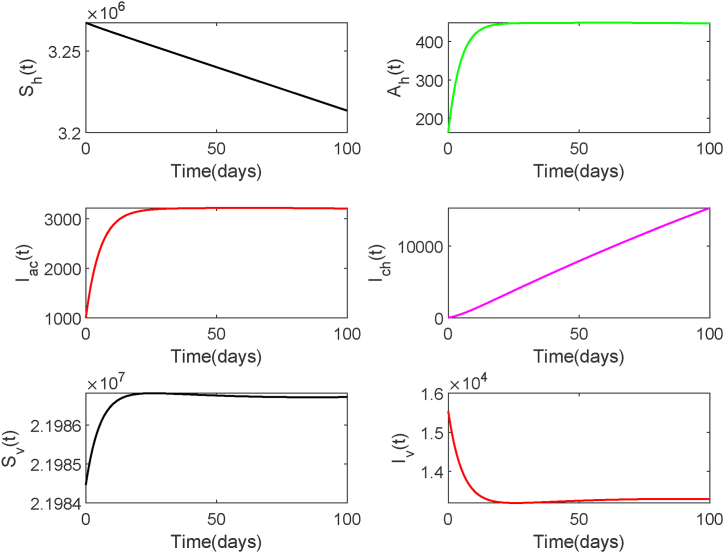
Solution Trajectories of system (2.1) when *R*_0_ > 1.

Compared with relevant Chikungunya studies, our estimate of *R*_0_ (0.40) in this study is slightly lower. For instance, González-Parra et al. (2019) calculated a range of 0.74–0.84 for Colombia's 2015 epidemic, while Feng et al. (2019) estimated an *R*_0_ of approximately 2.035 for the 2007 Chikungunya outbreak in Italy. In contrast, Li and Zhao (2024) obtained a significantly higher estimate of *R*_0_ = 3.6087 in Brazil. We primarily attribute this relatively low estimate to two main factors. First, the model is calibrated using data from the second phase of the outbreak (Zhang et al., 2025), during which intervention measures such as case isolation and public health education have already been implemented in Shunde District, significantly reducing transmission risk. Second, owing to its extensive experience with recurrent Dengue fever epidemics, Guangdong Province has established robust surveillance and control systems, which enabled the timely and effective containment of this Chikungunya outbreak. This finding underscores the critical importance of timely interventions in controlling outbreak dynamics.

## 4 Sensitivity analysis

### 4.1 Local sensitivity indices

This section employs local sensitivity methods to investigate the impact of parameter variations on *R*_0_ and to determine which parameters with significant influence. Sensitivity indices provide a quantitative measure of local parameter influence and are computed according to Zi (2011):

$$\gamma_{\xi }^{R_{0}}=\frac{\partial R_{0}}{\partial \xi }\frac{\xi }{R_{0}}.$$

The sensitivity indices are defined as follows for each parameter:

$$\begin{array}{ll} & \gamma_{B_{h}}^{R_{0}}=-\frac{1}{2},\;\gamma_{B_{v}}^{R_{0}}=\frac{1}{2},\;\gamma_{\beta }^{R_{0}}=1,\;\gamma_{\rho_{1}}^{R_{0}}=\frac{1}{2},\;\gamma_{\rho_{2}}^{R_{0}}=\frac{1}{2},\;\gamma_{\epsilon_{v}}^{R_{0}}=-1,\\ & \gamma_{p}^{R_{0}}=\frac{1}{2}\frac{p\left(\eta_{h}+\epsilon_{h}\right)-\kappa p\left(\sigma_{h}+\theta_{h}+\epsilon_{h}\right)}{p\left(\eta_{h}+\epsilon_{h}\right)+\kappa \left(1-p\right)\left(\sigma_{h}+\theta_{h}+\epsilon_{h}\right)},\\ & \gamma_{\eta_{h}}^{R_{0}}=-\frac{\eta_{h}}{2\left(\eta_{h}+\epsilon_{h}\right)}\frac{\kappa \left(1-p\right)\left(\sigma_{h}+\theta_{h}+\epsilon_{h}\right)}{p\left(\eta_{h}+\epsilon_{h}\right)+\kappa \left(1-p\right)\left(\sigma_{h}+\theta_{h}+\epsilon_{h}\right)},\\ & \gamma_{\sigma_{h}}^{R_{0}}=-\frac{\sigma_{h}}{2\left(\sigma_{h}+\theta_{h}+\epsilon_{h}\right)}\frac{p\left(\eta_{h}+\epsilon_{h}\right)}{p\left(\eta_{h}+\epsilon_{h}\right)+\kappa \left(1-p\right)\left(\sigma_{h}+\theta_{h}+\epsilon_{h}\right)},\\ & \gamma_{\theta_{h}}^{R_{0}}=-\frac{\theta_{h}}{2\left(\sigma_{h}+\theta_{h}+\epsilon_{h}\right)}\frac{p\left(\eta_{h}+\epsilon_{h}\right)}{p\left(\eta_{h}+\epsilon_{h}\right)+\kappa \left(1-p\right)\left(\sigma_{h}+\theta_{h}+\epsilon_{h}\right)},\\ & \gamma_{\kappa }^{R_{0}}=\frac{1}{2}\frac{\kappa \left(1-p\right)\left(\sigma_{h}+\theta_{h}+\epsilon_{h}\right)}{p\left(\eta_{h}+\epsilon_{h}\right)+\kappa \left(1-p\right)\left(\sigma_{h}+\theta_{h}+\epsilon_{h}\right)},\\ & \gamma_{\epsilon_{h}}^{R_{0}}=\frac{1}{2\left(\eta_{h}+\epsilon_{h}\right)\left(\sigma_{h}+\theta_{h}+\epsilon_{h}\right)}\frac{p\left(\sigma_{h}+\theta_{h}\right){\left(\eta_{h}+\epsilon_{h}\right)}^{2}+\kappa \left(1-p\right)\eta_{h}{\left(\sigma_{h}+\theta_{h}+\epsilon_{h}\right)}^{2}}{p\left(\eta_{h}+\epsilon_{h}\right)+\kappa \left(1-p\right)\left(\sigma_{h}+\theta_{h}+\epsilon_{h}\right)} \end{array}$$

Notably, a number of sensitivity indices exhibit dependence on one or more parameters, while the remaining indices are constants independent of parameter values. Table 4.6 lists their values calculated with the baseline parameters. Among these, the mosquito mortality rate *ϵ*_*v*_ has the greatest negative effect; conversely, the biting rate *β* has the strongest positive effect.

**Table 4.6:** Sensitivity indicators of *R*_0_.

Parameter	Index value	Parameter	Index value
*β*	1	p	0.286
*B*_*h*_	−0.5	*B*_*v*_	0.5
*ρ*_1_	0.5	*ρ*_2_	0.5
*σ*_*h*_	−0.153	*κ*	0.016
*ϵ*_*h*_	0.401	*ϵ*_*v*_	−1
*θ*_*h*_	−0.231	*η*_*h*_	−0.016

### 4.2 Global sensitivity analysis

Unlike local approaches, global sensitivity analysis evaluates simultaneous variations across a broader parameter space. This is accomplished by computing the Partial Rank Correlation Coefficient(PRCC) (Blower & Dowlatabadi, 1994; Marino et al., 2008) for each parameter, which are sampled using the Latin Hypercube Sampling (LHS) method.

Fig. 4.7 presents the PRCC analysis result for *R*_0_, revealing that parameters *β*, *ρ*_1_, *ρ*_2_, *p*, *ϵ*_*h*_, *κ* and *B*_*v*_ are positively correlated with *R*_0_, while *B*_*h*_, *η*_*h*_, *σ*_*h*_, *θ*_*h*_ and *ϵ*_*v*_ are negatively correlated with *R*_0_. The analysis identifies the mosquito biting rate (*β*) and mortality rate (*ϵ*_*v*_) as the dominant influences on *R*_0_, exhibiting strongly positive and negative effects, respectively. The PRCC values for both parameters close to 1, indicating that they have a highly significant influence on *R*_0_.

**Fig. 7:**
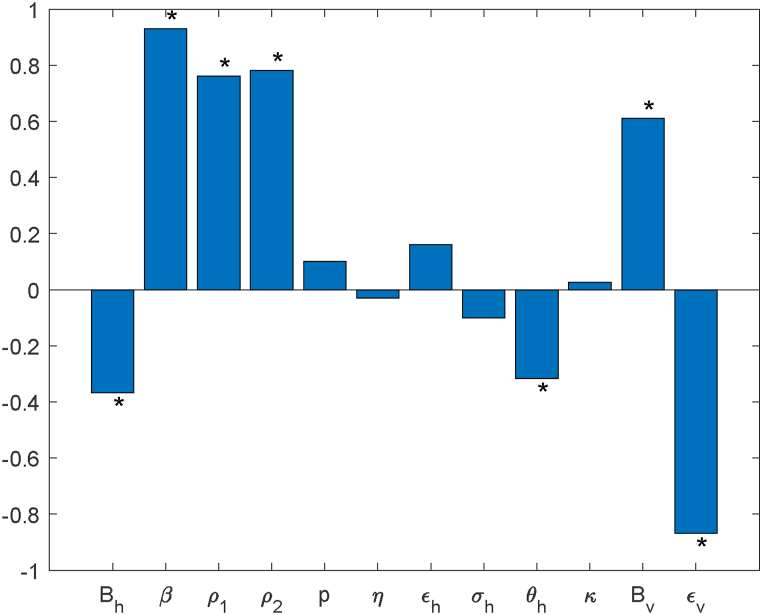
PRCC of *R*_0_.

Both local and global sensitivity analyses demonstrate that the model output is most sensitive to the mosquito biting rate (*β*) and mortality rate (*ϵ*_*v*_). This result indicates that reducing the mosquito biting rate and enhancing the mosquito mortality rate are two key intervention strategies for controlling the spread of Chikungunya fever. The findings not only clarify the priority directions for prevention and control measures, but also provide a direct basis for the introduction of time-dependent intervention measures in the subsequent optimal control analysis.

## 5 Optimal control

In response to the prevalence of Chikungunya fever in Foshan City, Shunde District has established a multi-level prevention and control system centered on “surveillance and early warning—blocking transmission sources—comprehensive vector management”. The system first enhances case screening in medical institutions and dynamic monitoring of mosquito vector density to achieve early warning. It also enforces mosquito-proof isolation of confirmed cases and ensures prompt management of infection hotspots. For vector intervention, a strategy emphasizing “environmental management as the primary approach, supplemented by chemical control” is adopted. This involves mobilizing the public to eliminate breeding sites and integrating the use of biological and chemical larvicides along with adult mosquito control measures. Furthermore, the system promotes public health education, establishes a multi-sectoral joint prevention and control mechanism, and implements risk-based zoning and graded management strategies.

Building upon this framework and considering the results of sensitivity analysis, we identify parameters *β* and *ϵ*_*v*_ as the dominant factors influencing the transmission of Chikungunya fever. Accounting for the management needs of chronic cases, this study aims to present a dynamic optimal control framework to optimize the allocation and scheduling of limited public health resources among key intervention measures in Shunde District. Therefore, the following five time-varying control measures are introduced:

• *u*_1_(*t*): Enhance public awareness campaigns to disseminate knowledge on prevention and control, urging all residents to adopt personal safeguard strategies such as donning long-sleeved garments and employing mosquito netting, and installing window screens to reduce mosquito bites;
• *u*_2_(*t*): Screen for asymptomatic infections; once identified, isolate such individuals. Since asymptomatic individuals show no apparent adverse symptoms, isolation prevents them from participating in the transmission process;
• *u*_3_(*t*): For acute infections, adopt interventions including acupoint patches, topical medicinal applications, and traditional Chinese medicine treatments to shorten the duration of the acute phase and alleviate symptoms such as fever, joint pain, and rash;
• *u*_4_(*t*): Strengthen treatment for chronic infections to mitigate joint symptoms and shorten the chronic phase;
• *u*_5_(*t*): Implement mosquito eradication campaigns to achieve comprehensive vector elimination.

The following system represents the model incorporating combined intervention strategies:

(5.5) $$\left\{\begin{array}{l} \frac{dS_{h}}{dt}=B_{h}-\frac{\left(1-u_{1}\left(t\right)\right)\beta \rho_{1}I_{v}S_{h}}{T_{h}}-\epsilon_{h}S_{h},\;\\ \frac{dA_{h}}{dt}=\frac{\left(1-u_{1}\left(t\right)\right)\beta \rho_{1}\left(1-p\right)I_{v}S_{h}}{T_{h}}-\left(\eta_{h}+u_{2}\left(t\right)\right)A_{h}-\epsilon_{h}A_{h},\;\\ \frac{dI_{ac}}{dt}=\frac{\left(1-u_{1}\left(t\right)\right)\beta \rho_{1}pI_{v}S_{h}}{T_{h}}-\left(1+u_{3}\left(t\right)\right)\sigma_{h}I_{ac}-\left(1+u_{3}\left(t\right)\right)\theta_{h}I_{ac}-\epsilon_{h}I_{ac},\;\\ \frac{dI_{ch}}{dt}=\left(1+u_{3}\left(t\right)\right)\sigma_{h}I_{ac}-\left(1+u_{4}\left(t\right)\right)\delta_{h}I_{ch}-\epsilon_{h}I_{ch},\;\\ \frac{dR_{h}}{dt}=\left(\eta_{h}+u_{2}\left(t\right)\right)A_{h}+\left(1+u_{3}\left(t\right)\right)\theta_{h}I_{ac}+\left(1+u_{4}\left(t\right)\right)\delta_{h}I_{ch}-\epsilon_{h}R_{h},\;\\ \frac{dS_{v}}{dt}=B_{v}-\frac{\left(1-u_{1}\left(t\right)\right)\beta \rho_{2}\left(I_{ac}+\kappa A_{h}\right)S_{v}}{T_{h}}-\left(\epsilon_{v}+u_{5}\left(t\right)\right)S_{v},\;\\ \frac{dI_{v}}{dt}=\frac{\left(1-u_{1}\left(t\right)\right)\beta \rho_{2}\left(I_{ac}+\kappa A_{h}\right)S_{v}}{T_{h}}-\left(\epsilon_{v}+u_{5}\left(t\right)\right)I_{v}.\; \end{array}\right)$$

The five control measures are denoted by *U*(*t*) = (*u*_1_(*t*), *u*_2_(*t*), *u*_3_(*t*), *u*_4_(*t*), *u*_5_(*t*)). The goal of optimal control is to determine a set of optimal controls *U*∗(*t*) that minimizes the number of infected individuals (including asymptomatic, acute, and chronic cases), while also minimizing the implementation costs. Accordingly, the corresponding objective function takes the form:

$$J\left(U\right)=\int_{0}^{t}L\left(A_{h},I_{ac},I_{ch},U\left(t\right)\right)\;dt,$$

where the integrand is given by $L\left(A_{h},I_{ac},I_{ch},U\left(t\right)\right)=K_{1}A_{h}+K_{2}I_{ac}+K_{3}I_{ch}+\sum_{i=1}^{5}\frac{1}{2}T_{i}u_{i}^{2}\left(t\right)$. Here *K*_1_, *K*_2_ and *K*_3_ represent the weighting coefficients for the asymptomatic (*A*_*h*_), acute (*I*_*ac*_), and chronic (*I*_*ch*_) cases, respectively; while *T*_*i*_(*i* = 1, *…*, 5) quantify the implementation costs of the five control measures *u*_*i*_(*t*).

Theorem 5.1 *For the control model* 5.5*, there exists an optimal control* $U^{*}=\left(u_{1}^{*},u_{2}^{*},u_{3}^{*},u_{4}^{*},u_{5}^{*}\right)$ *such that*

$$J\left(U^{*}\right)=minJ\left(u_{1},u_{2},u_{3},u_{4},u_{5}\right).$$

Proof. For an optimal control to exist, the following criteria must be satisfied: (1) According to Lukes’ result (Lukes, 1982), all control and state variables take on non-negative values; (2) The admissible control set *U* is closed and convex; (3) The system is bounded, ensuring the compactness required for the existence proof. (4) Since the integrand is a quadratic function in the control variables, the objective functional exhibits convexity over the admissible set *U*; (5) Moreover, owing to the fact that

$$\begin{array}{ll} & \;\\ & K_{1}A_{h}+K_{2}I_{ac}+K_{3}I_{ch}+\frac{1}{2}T_{1}u_{1}^{2}\left(t\right)+\frac{1}{2}T_{2}u_{2}^{2}\left(t\right)+\frac{1}{2}T_{3}u_{3}^{2}\left(t\right)+\frac{1}{2}T_{4}u_{4}^{2}\left(t\right)+\frac{1}{2}T_{5}u_{5}^{2}\left(t\right)\\ \geq & \frac{1}{2}T_{1}u_{1}^{2}\left(t\right)+\frac{1}{2}T_{2}u_{2}^{2}\left(t\right)+\frac{1}{2}T_{3}u_{3}^{2}\left(t\right)+\frac{1}{2}T_{4}u_{4}^{2}\left(t\right)+\frac{1}{2}T_{5}u_{5}^{2}\left(t\right)\\ \geq & \frac{1}{2}\min\left\{T_{1},T_{2},T_{3},T_{4},T_{5}\right\}\sum_{i=1}^{5}u_{i}^{2}\left(t\right). \end{array}$$

We can find constants *b* > 1, *a*_1_ > 0, and *a*_2_ > 0 satisfying:

$$\begin{array}{ll} & \;\\ & K_{1}A_{h}+K_{2}I_{ac}+K_{3}I_{ch}+\frac{1}{2}T_{1}u_{1}^{2}\left(t\right)+\frac{1}{2}T_{2}u_{2}^{2}\left(t\right)+\frac{1}{2}T_{3}u_{3}^{2}\left(t\right)+\frac{1}{2}T_{4}u_{4}^{2}\left(t\right)+\frac{1}{2}T_{5}u_{5}^{2}\left(t\right)\\ \geq & a_{1}{\left(|u_{1}{|}^{2}+|u_{2}{|}^{2}+|u_{3}{|}^{2}+|u_{4}{|}^{2}+|u_{5}{|}^{2}\right)}^{\frac{b}{2}}+a_{2}. \end{array}$$

Theorem 5.2 *Let* $u^{*}=\left(u_{1}^{*}\left(t\right),u_{2}^{*}\left(t\right),u_{3}^{*}\left(t\right),u_{4}^{*}\left(t\right),u_{5}^{*}\left(t\right)\right)$ *be the optimal control,* $\left({\bar{S}}_{h},{\bar{A}}_{h},{\bar{I}}_{ac},{\bar{I}}_{ch},{\bar{R}}_{h},{\bar{S}}_{v},{\bar{I}}_{v}\right)$ *be the optimal state solution. We formulate the Hamiltonian function:*

$$H\left(X,U,\lambda ,t\right)=L\left(A_{h},I_{ac},I_{ch},U\left(t\right)\right)+\overset{7}{\underset{i=1}{\sum }}\lambda_{i}\frac{dx_{i}}{dt}.$$

There are adjoint variables λ(t) = (λ_1_(t), λ_2_(t), λ_3_(t), λ_4_(t), λ_5_(t), λ_6_(t), λ_7_(t)) satisfying

$$\begin{array}{ll} \frac{d\lambda_{1}}{dt} & =-\frac{\partial H}{\partial S_{h}}\\ & =\lambda_{1}\left(\frac{\left(1-u_{1}\right)\beta \rho_{1}I_{v}}{T_{h}}+\epsilon_{h}\right)-\lambda_{2}\frac{\left(1-p\right)\left(1-u_{1}\right)\beta \rho_{1}I_{v}}{T_{h}}-\lambda_{3}\frac{p\left(1-u_{1}\right)\beta \rho_{1}I_{v}}{T_{h}},\\ \frac{d\lambda_{2}}{dt} & =-\frac{\partial H}{\partial V_{h}}\\ & =-K_{1}-\lambda_{2}\left(\eta +u_{2}+\epsilon_{h}\right)-\lambda_{5}\left(\eta +u_{2}\right)+\lambda_{6}\frac{\left(1-u_{1}\right)\beta \rho_{2}\kappa S_{v}}{T_{h}}-\lambda_{7}\frac{\left(1-u_{1}\right)\beta \rho_{2}\kappa S_{v}}{T_{h}},\\ \frac{d\lambda_{3}}{dt} & =-\frac{\partial H}{\partial I_{ac}}\\ & =-K_{2}+\lambda_{3}\left(\left(1+u_{3}\right)\sigma_{h}+\left(1+u_{3}\right)\theta_{h}+\epsilon_{h}\right)-\lambda_{4}\left(1+u_{3}\right)\sigma_{h}-\lambda_{5}\left(1+u_{3}\right)\theta_{h}+\lambda_{6}\frac{\left(1-u_{1}\right)\beta \rho_{2}S_{v}}{T_{h}}-\lambda_{7}\frac{\left(1-u_{1}\right)\beta \rho_{2}S_{v}}{T_{h}}, \end{array}$$

$$\begin{array}{ll} \frac{d\lambda_{4}}{dt} & =-\frac{\partial H}{\partial I_{ch}}\\ & =-K_{3}+\lambda_{4}\left(\left(1+u_{4}\right)\delta_{h}+\epsilon_{h}\right)-\lambda_{5}\left(1+u_{4}\right)\delta_{h},\\ \frac{d\lambda_{5}}{dt} & =-\frac{\partial H}{\partial R_{h}}=\lambda_{5}\epsilon_{h},\\ \frac{d\lambda_{6}}{dt} & =-\frac{\partial H}{\partial S_{v}}\\ & =\lambda_{6}\left(\frac{\left(1-u_{1}\right)\beta \rho_{2}\left(I_{ac}+\kappa A_{h}\right)}{T_{h}}+\epsilon_{v}+u_{5}\right)-\lambda_{7}\frac{\left(1-u_{1}\right)\beta \rho_{2}\left(I_{ac}+\kappa A_{h}\right)}{T_{h}}，\\ \frac{d\lambda_{7}}{dt} & =-\frac{\partial H}{\partial I_{v}}\\ & =\lambda_{1}\frac{\left(1-u_{1}\right)\beta \rho_{1}S_{h}}{T_{h}}-\lambda_{2}\frac{\left(1-p\right)\left(1-u_{1}\right)\beta \rho_{1}S_{h}}{T_{h}}-\lambda_{3}\frac{p\left(1-u_{1}\right)\beta \rho_{1}S_{h}}{T_{h}}+\lambda_{7}\left(\epsilon_{v}+u_{5}\right). \end{array}$$

With the transversality conditions given by λ_i_(T) = 0(i = 1, …, 7), the resulting optimality condition for the control is:

$$\begin{array}{ll} u_{1}^{*} & =\min\left\{1,\max\left\{0,\frac{\beta \rho_{1}I_{v}S_{h}}{T_{h}T_{1}}\left(\lambda_{2}\left(1-p\right)+\lambda_{3}p-\lambda_{1}\right)+\frac{\beta \rho_{2}\left(I_{ac}+\kappa A_{h}\right)S_{v}}{T_{h}T_{1}}\left(\lambda_{7}-\lambda_{6}\right)\right\}\right\},\\ u_{2}^{*} & =\min\left\{1,\max\left\{0,\frac{\left(\lambda_{2}-\lambda_{5}\right)A_{h}}{T_{2}}\right\}\right\},\\ u_{3}^{*} & =\min\left\{1,\max\left\{0,\frac{\left(\lambda_{3}-\lambda_{4}\right)\sigma_{h}I_{ac}}{T_{3}}+\frac{\left(\lambda_{3}-\lambda_{5}\right)\theta_{h}I_{ac}}{T_{3}}\right\}\right\},\\ u_{4}^{*} & =\min\left\{1,\max\left\{0,\frac{\left(\lambda_{4}-\lambda_{5}\right)\delta_{h}I_{ch}}{T_{4}}\right\}\right\},\\ u_{5}^{*} & =\min\left\{1,\max\left\{0,\frac{\lambda_{6}S_{v}+\lambda_{7}I_{v}}{T_{5}}\right\}\right\}. \end{array}$$

Proof. Following Pontryagin's maximum principle (Pontryagin, 2018) for the defined Hamiltonian, we obtain the adjoint variable vector $\lambda \left(t\right)\in R^{7}$. The adjoint system can be derived through $\frac{d\lambda_{i}}{dt}=-\frac{\partial H}{\partial X_{i}}$. By applying the necessary conditions for optimality, we obtain:

$$\begin{array}{ll} \frac{\partial H}{\partial u_{1}} & =T_{1}u_{1}\left(t\right)-\lambda_{1}\frac{\beta \rho_{1}I_{v}S_{h}}{T_{h}}-\lambda_{2}\frac{\left(1-p\right)\beta \rho_{1}I_{v}S_{h}}{T_{h}}-\lambda_{3}\frac{p\beta \rho_{1}I_{v}S_{h}}{T_{h}}+\lambda_{6}\frac{\beta \rho_{2}\left(I_{ac}+\kappa A_{h}\right)S_{v}}{T_{h}}-\lambda_{7}\frac{\beta \rho_{2}\left(I_{ac}+\kappa A_{h}\right)S_{v}}{T_{h}},\\ \frac{\partial H}{\partial u_{2}} & =T_{2}u_{2}\left(t\right)+\lambda_{1}A_{h}+\lambda_{5}A_{h},\\ \frac{\partial H}{\partial u_{3}} & =T_{3}u_{3}\left(t\right)-\lambda_{3}\left(\sigma_{h}I_{ac}+\theta_{h}I_{ac}\right)+\lambda_{4}\sigma_{h}I_{ac}+\lambda_{5}\theta_{h}I_{ac},\\ \frac{\partial H}{\partial u_{4}} & =T_{4}u_{4}\left(t\right)-\lambda_{4}\delta_{h}I_{ch}+\lambda_{5}\delta_{h}I_{ch},\\ \frac{\partial H}{\partial u_{5}} & =T_{5}u_{5}\left(t\right)-\lambda_{6}S_{v}+\lambda_{7}I_{v}. \end{array}$$

The optimal control solutions are obtained by solving $\frac{\partial H}{\partial u_{i}}=0\left(i=1,2,3,4,5\right)$:

$$\begin{array}{ll} u_{1}\left(t\right) & =\frac{\beta \rho_{1}I_{v}S_{h}}{T_{h}T_{1}}\left(\lambda_{2}\left(1-p\right)+\lambda_{3}p-\lambda_{1}\right)+\frac{\beta \rho_{2}\left(I_{ac}+\kappa A_{h}\right)S_{v}}{T_{h}T_{1}}\left(\lambda_{7}-\lambda_{6}\right),\\ u_{2}\left(t\right) & =\frac{\left(\lambda_{2}-\lambda_{5}\right)A_{h}}{T_{2}},\\ u_{3}\left(t\right) & =\frac{\left(\lambda_{3}-\lambda_{4}\right)\sigma_{h}I_{ac}}{T_{3}}+\frac{\left(\lambda_{3}-\lambda_{5}\right)\theta_{h}I_{ac}}{T_{3}},\\ u_{4}\left(t\right) & =\frac{\left(\lambda_{4}-\lambda_{5}\right)\delta_{h}I_{ch}}{T_{4}},\\ u_{5}\left(t\right) & =\frac{\lambda_{6}S_{v}+\lambda_{7}I_{v}}{T_{6}}. \end{array}$$

## 6 Numerical simulations

### 6.1 Control strategy simulation

To evaluate the static effect of fixed-intensity intervention measures, we assigned three distinct sets of constant values to the control measures *U*: (1) No control: *u*_*i*_ = 0 for *i* = 1, *…*, 5; (2)Moderate control: *u*_1_ = *u*_2_ = *u*_3_ = 0.2, *u*_4_ = 0.15, *u*_5_ = 0.1; (3)Strong control: *u*_1_ = *u*_2_ = *u*_3_ = 0.5, *u*_4_ = 0.3, *u*_5_ = 0.2. Under varying control intensities, Fig. 6.8 illustrates the comparative trajectories of human infection states and infected mosquito populations.

**Fig. 8:**
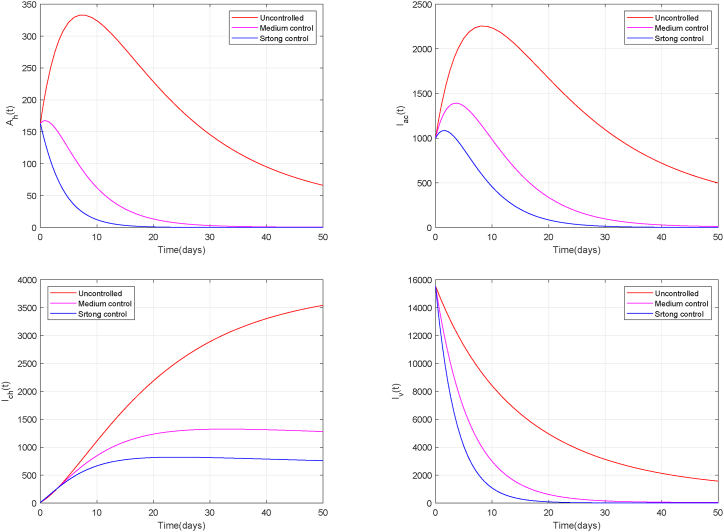
Population dynamics of infected subpopulations under constant control.

As shown in Fig. 6.8, increasing the control intensity leads to a marked bidirectional suppression effect within the human–mosquito Chikungunya transmission system. In the uncontrolled scenario, asymptomatic and acute human infections attain peaks of 332 and 2252, respectively. Under moderate control intensity, these peaks decline to 167 and 1392, with substantial reductions also observed in the number of chronic infections and infected mosquitoes. With intervention intensity further increased to a strong level, all infected human compartments and infected mosquito continue to decline from the medium control baseline, but the extent of reduction is less pronounced. This indicates that the declining trend does not follow a simple linear relationship with increasing control intensity.

Constant control represents a simplified abstraction of practical disease control measures, with its outcomes serving as a benchmark for evaluating more complex dynamic control strategies. We next consider the optimal control strategy. To explore temporal allocation of dynamically optimized interventions, we employ a forward-backward sweep algorithm that relies on the Runge-Kutta (Lenhart & Workman, 2007) numerical scheme within the MATLAB environment. In the numerical experiments, the control period is set to 50 days, with *K*_1_ = 30, *K*_2_ = 40, *K*_3_ = 20, *T*_1_ = 500, *T*_2_ = 900, *T*_3_ = 1000, *T*_4_ = 1500, *T*_5_ = 1600. The cost coefficients are assigned based on the complexity and resource intensity of each intervention, with the specific values being hypothetical. This costing structure captures their relative resource use. Public health education, focused on information dissemination, was assigned the lowest value. Asymptomatic screening, requiring testing kits and personnel, received a moderate value. Treatments for acute and chronic patients involve healthcare resources, with chronic treatment incurring greater expense due to its prolonged nature. Comprehensive mosquito spraying is assigned the highest cost, as it demands chemicals, equipment, extensive labor, and accounts for ecological expenses.

First, we consider Strategy A (*u*_1_, *u*_4_, *u*_5_), which emphasizes personal protection to reduce mosquito bites, comprehensive mosquito elimination, and chronic cases management. This strategy primarily focuses on infection prevention measures. As shown in Fig. 6.9, under the implementation of preventive measures such as avoiding mosquito bites and vector elimination, the peaks of asymptomatic and acute infections are significantly reduced, and the decay rate of infected mosquitoes increases. Furthermore, with the addition of chronic case management, the number of chronic infections also decreases substantially. This outcome aligns with the public health principle that prevention is superior to treatment. The final graph in Fig. 6.9 displays the time-varying profiles of the control measures. Both *u*_1_ and *u*_5_ are deployed at peak intensity for initial periods of 26 and 30 days, respectively, before a gradual reduction. In contrast, the chronic case management measure *u*_4_ reaches its peak around day 4, slightly later than the other two controls.

**Fig. 9:**
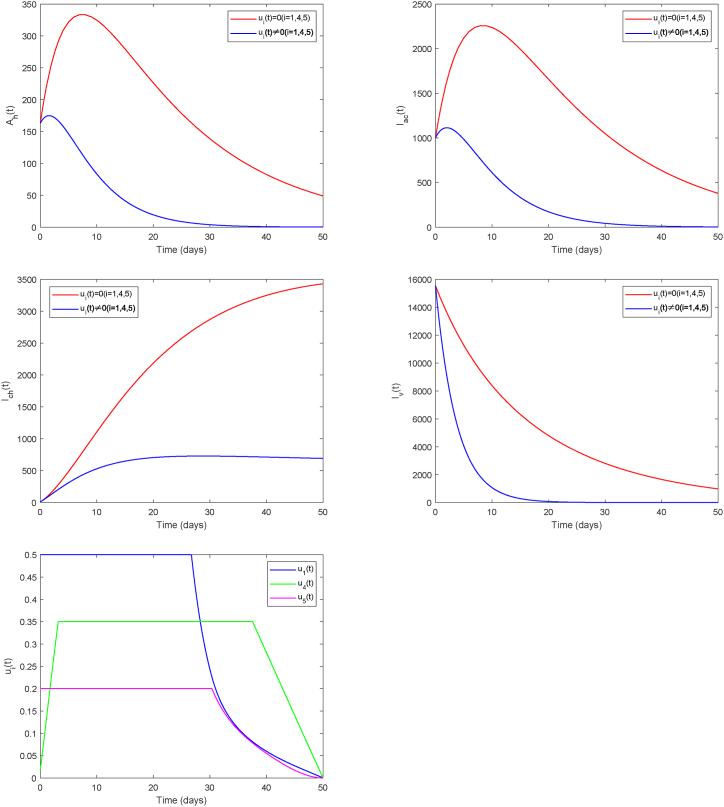
Strategy A

**Fig. 10:**
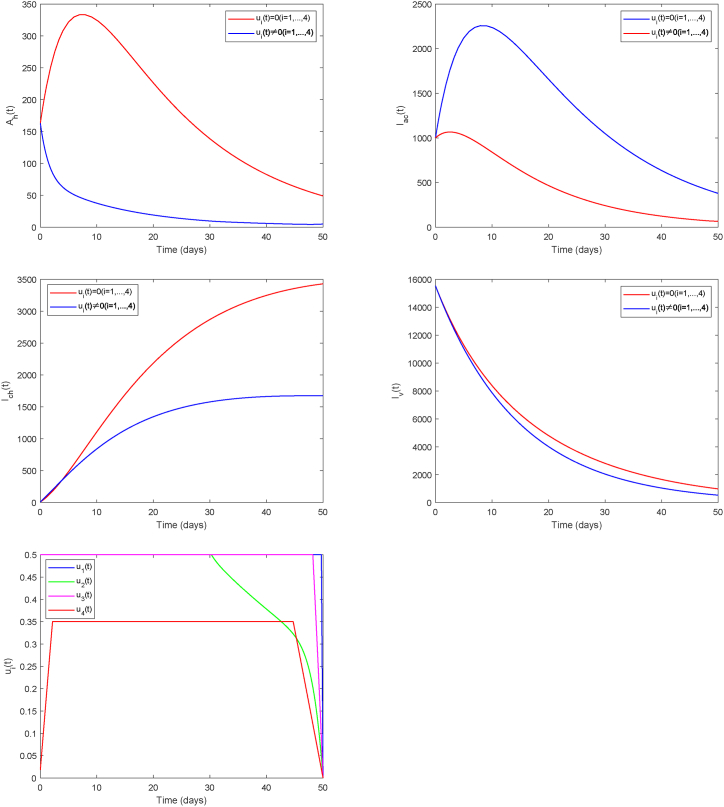
Strategy B.

We now focus on Strategy B (*u*_1_, *u*_2_, *u*_3_, *u*_4_), which emphasizes mosquito-bite prevention, medical testing, and treatment, to evaluate its effectiveness and cost in reducing the spread of Chikungunya (see Fig. 10). Findings confirm that the strategy effectively reduced the peak and overall scale of human infections. However, its suppressive effect on infected mosquitoes is not significant. Additionally, all control measures need to be implemented at maximum intensity for an extended duration, resulting in relatively high costs. This suggests that relying solely on physical protection and medical management, without mosquito elimination measures, still poses certain challenges in controlling the spread of Chikungunya.

**Fig. 11:**
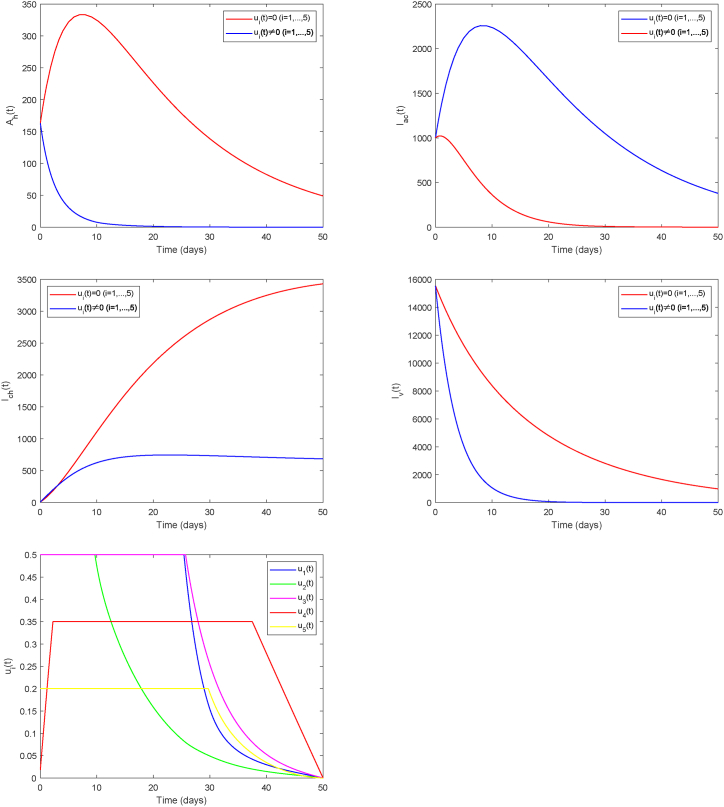
Strategy C.

Therefore, we further incorporate comprehensive mosquito elimination into the strategy, denoted as Strategy C (*u*_1_, *u*_2_, *u*_3_, *u*_4_, *u*_5_). A key benefit of the strategy is the drastic reduction in the high-intensity control period, achieved through its dual impact: a sharp decline in human infections and a markedly accelerated decay of the infected mosquito population, leading to more effective disease containment (see Fig. 11). These findings underscore the importance of mosquito elimination measures in the containment of Chikungunya transmission.

**Fig. 12:**
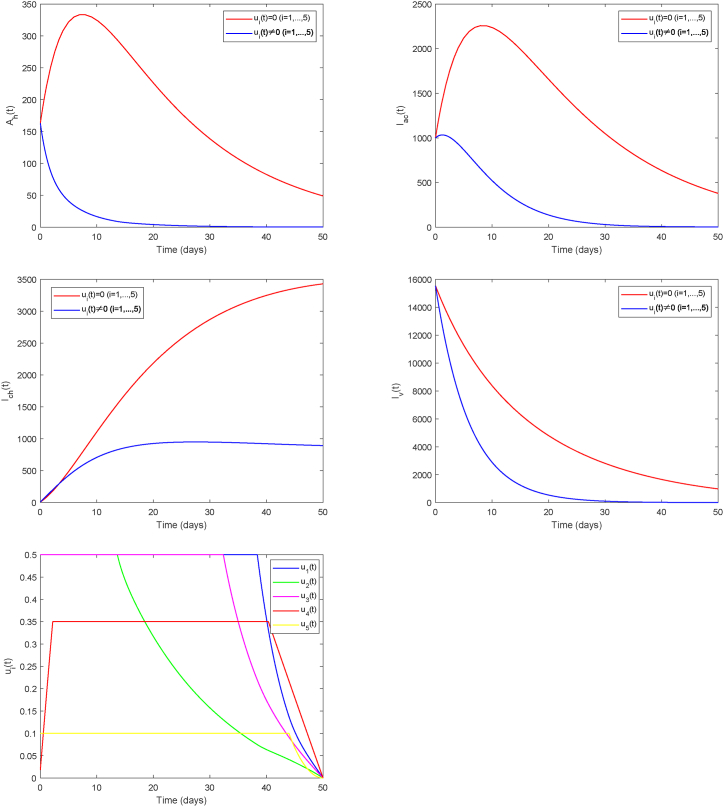
Strategy D

Similarly, building upon Strategy C, we further adjust the approach by reducing the maximum intensity of comprehensive mosquito elimination by half, resulting in an integrated control Strategy D (*u*_1_, *u*_2_, *u*_3_, *u*_4_, *u*_5_). This adjustment addresses environmental concerns from overusing control agents, and to evaluate how intervention intensity influences overall efficacy. It can be observed that reducing the mosquito elimination intensity leads to a prolonged duration of other control measures being applied at their maximum strength (see Fig. 12). While this lowers environmental cost, it increases the total control expenditure. Hence, in practical implementation, appropriately raising the intensity of mosquito control could not only reduce overall costs but also rapidly achieve more effective suppression of Chikungunya transmission. Such a strategy will help balance control efficacy, economic cost, and environmental impact.

### 6.2 Cost-effectiveness analysis

When evaluating the practical value of disease prevention and control measures, focusing solely on their efficacy in containing disease spread does not suffice to form a complete basis for decision-making. The implementation of any control measure inevitably requires corresponding resource allocation. Excessively high costs may not only hinder the feasibility of interventions but also divert resources from other public health priorities, thereby compromising overall control effectiveness. Therefore, both the effectiveness and economic efficiency of control measures must be considered. We employ the Incremental Cost-Effectiveness Ratio (ICER) (Okosun et al., 2013) to further quantify investment costs and resulting benefits of different control strategies, which is calculated as follows:

$$ICER=\frac{\text{Cost of Strategy B}-\text{Cost of Strategy A}}{\text{Infections Averted by Strategy B}-\text{Infections Averted by Strategy A}}.$$

The strategy cost is computed by the integral: $\int_{0}^{t_{c}}\left(T_{1}u_{1}+T_{2}u_{2}+T_{3}u_{3}+T_{4}u_{4}+T_{5}u_{5}\right)dt$, reflecting the cumulative expenditure across all control measures over the entire period. We compute infections averted by the strategy as: $\int_{0}^{t_{c}}\left(I\left(t\right)-\tilde{I}\left(t\right)\right)dt$, where *I*(*t*) denotes infections without intervention and $\tilde{I}\left(t\right)$ represents infections under the corresponding control strategy. Table 6.7 summarizes the model simulation results, presenting strategies ranked by ascending infections averted alongside their computed ICER values.

**Table 6.7:** Cost-effectiveness comparison of strategies A-D

Strategy	Infection Averted	Cost	ICER	Cost of Chonic Case Management
B	43465.16	81325.62	1.87	24333.20
A	51826.87	41969.84	−4.71	22186.76
D	53700.76	72158.44	16.11	23162.24
C	56509.87	65210.89	−2.47	22400.98

According to the computational results presented in Table 6.7, both ICER(A) and ICER(C) are negative. Comparing Strategy A with Strategy B, and Strategy C with Strategy D, it is evident that Strategy A and Strategy C incur relatively lower costs while averting a larger number of infections, demonstrating superior effectiveness and cost-effectiveness. Therefore, only Strategy A and Strategy C need to be compared in the subsequent analysis.

As clearly shown in Table 6.8, ICER(A) < ICER(C), indicating that Strategy C incurs higher costs while yielding inferior containment effectiveness. It can therefore be concluded that Strategy A achieves the highest cost-effectiveness. In other words, under resource constraints, implementing control measures such as personal protection and mosquito elimination can not only effectively curb the transmission of Chikungunya fever but also reduce the overall cost of control efforts. A comparison of the costs of chronic case management across the various strategies reveals that Strategy A required the lowest expenditure. From the perspective of mitigating long-term chronic disease burden, it also proves to be a highly favorable strategy.

**Table 6.8:** Cost-effectiveness comparison of strategies A and C.

Strategy	Infection Averted	Cost	ICER	Cost of Chonic Case Management
A	51826.87	41969.84	0.81	22186.76
C	56509.87	65210.89	4.96	22400.98

## 7 Conclusion and discussions

In response to the Chikungunya outbreak in Foshan City, this paper establishes a cross-infection differential equation model between humans and mosquitoes that incorporates a chronic infection compartment in the human population. We analysis the system's dynamics and derive optimal control strategies, yielding the following primary findings:

Firstly, an explicit expression for *R*_0_ is obtained. Stability analysis demonstrates that when *R*_0_ < 1, the disease-free equilibrium is globally asymptotically stable, leading to disease extinction. Conversely, if *R*_0_ > 1, the endemic equilibrium is globally asymptotically stable, indicating that the Chikungunya epidemic may persist. These results are further validated through numerical simulations.

Secondly, the model parameters are fitted using the daily reported Chikungunya case data from Shunde District, yielding a good fit (*R*^2^ = 0.76). Key transmission parameters are successfully quantified: the mosquito biting rate is estimated as *β* = 0.2111, the human infection probability per bite as *ρ*_1_ = 0.1932, the mosquito infection probability as *ρ*_2_ = 0.1118, the total mosquito population as *T*_*v*_ = 6071859, and the initial infected vector count as *I*_*v*_(0) = 15536. The constructed model accurately captures the actual transmission dynamics of the Chikungunya virus in Shunde District. Although some deviation between the observed data and model predictions is observed during the validation period, this discrepancy is explainable and intuitively demonstrates the success of intervention measures for disease containment. The basic reproduction number, calculated from the fitted parameters, is *R*_0_ = 0.40. This indicates a relatively low transmission potential of Chikungunya in Shunde District, which is consistent with the observed downward trend in daily cases. It also reflects the significant impact of timely mosquito control and prevention measures implemented by local authorities since the emergence of the outbreak.

Subsequently, local and global sensitivity analyses reveal that parameters including mosquito biting rate (*β*) and mosquito mortality rate (*ϵ*_*v*_) exert a pronounced influence on *R*_0_. This finding underscores that interventions targeting mosquito vectors represent the most efficient approach for controlling Chikungunya outbreaks, thereby providing a theoretical foundation for the formulation of preventive measures.

Finally, incorporating the containment measures implemented in Shunde District, this study introduces five intervention strategies: personal protection, screening and testing, treatment of acute cases, treatment of chronic cases, and comprehensive mosquito vector eradication, to simulate their containment effectiveness against the transmission of Chikungunya. Analysis under both constant control and optimal control frameworks indicates that higher control intensities lead to better suppression of Chikungunya spread, though the relationship is not purely linear. Beyond a certain level of control intensity, the marginal gain in effectiveness diminishes, while the associated costs continue to rise. The optimal control strategy A is dominant in terms of cost-effectiveness, implementing this strategy can effectively reduce the peak number of acute infections by 50.69 % while also mitigating the potential long-term burden of chronic diseases. The results further emphasize that prioritizing preventive measures aimed at curtailing mosquito density and human-vector contact rates can rapidly contain outbreaks, lower the transmission risk of Chikungunya, and optimize short-term cost efficiency.

This study conducts parameter fitting based on the Chikungunya outbreak in Shunde District, Foshan City, thereby enhancing the regional specificity and practical guidance of the conclusions. Through the analysis of the model fitting presented earlier, we have also identified objectives for future work. For instance, the model could be further refined by incorporating seasonal dynamics, integrating local meteorological and vector surveillance data to enable coupled analysis of long-term climate change impacts. Additionally, efforts could focus on parameterizing external suppression factors, such as correlating the mosquito mortality rate (*ϵ*_*v*_) with vector control intensity data, to develop a next-generation model that dynamically responds to public health interventions and exhibits enhanced predictive capability. Beyond these, an impulsive control strategy for mosquito elimination could be introduced to quantitatively simulate the effects of Foshan's vector control campaigns.

## CRediT authorship contribution statement

**Yan Wang:** Writing – review & editing, Writing – original draft, Supervision, Software, Methodology, Investigation, Conceptualization. **Huan Ma:** Software, Data curation, Conceptualization. **Qian Yan:** Validation, Methodology, Formal analysis. **Zhichun Yang:** Writing – review & editing, Supervision, Investigation, Conceptualization.

## Funding support

This work was supported by the 10.13039/501100001809National Natural Science Foundation of China (Nos. 12101513, 12471153 and 1197108), 10.13039/100010338Chongqing Normal University Foundation Program (No. 24XLB001), Project of Science and Technology Research Program of 10.13039/501100007957Chongqing Municipal Education Commission (No. KJQN202500513), Chongqing Talents Program (No. cstc2024ycjh-bgzxm0046), Higher Education Research Planning Project of China Association of Higher Education (No. 24SX0305), the “Unveiling and Leading” Project of Chongqing Municipal Commission of Economy and Information Technology (No. YJX-2025001001004).

## Declaration of competing interest

The authors declare that they have no known competing financial interests or personal relationships that could have appeared to influence the work reported in this paper.
